# Mosaic of plesiomorphic and derived characters in an Eocene myliobatiform batomorph (Chondrichthyes, Elasmobranchii) from Italy defines a new, basal body plan in pelagic stingrays

**DOI:** 10.1186/s40851-019-0128-0

**Published:** 2019-04-25

**Authors:** Giuseppe Marramà, Giorgio Carnevale, Gavin J. P. Naylor, Jürgen Kriwet

**Affiliations:** 1 Department of Palaeontology, University of Vienna, Althanstrasse 14, 1090 Vienna, Austria; 2 Dipartimento di Scienze della Terra, Università degli Studi di Torino, Via Valperga Caluso 35, 10125 Torino, Italy; 3 Florida Museum of Natural History, University of Florida, 1659 Museum Road, Gainesville, 32611 USA

**Keywords:** *Promyliobatis gazolai*, Myliobatiformes, Ypresian, Bolca Konservat-Lagerstätte, Phylogeny

## Abstract

**Background:**

End-Cretaceous niche-filling by benthic Mesozoic survivors resulted in a prominent increase of durophagous fish families, resulting in the appearance of the earliest representatives of several extant fish lineages, including the pelagic durophagous stingrays, a monophyletic clade of myliobatiform batoids that is characterized by a derived swimming mode and feeding habits. Although the earliest members appeared in the Late Cretaceous, most of the crown genera date back to the Eocene.

**Results:**

In this study, we re-examine the anatomy of the Eocene eagle ray *Promyliobatis gazolai* (de Zigno), represented by two nearly complete and articulated specimens from the world-famous Ypresian Konservat-Lagerstätte of Bolca, in detail. This taxon exhibits a mosaic of plesiomorphic and derived characters (e.g. tail sting displaced posteriorly on the tail, at about 50–60% of tail length; pectoral fins joining in front of the head; anterior and posterior pectoral fin margins nearly straight; compagibus laminam absent; single, unfragmented mesopterygium) that clearly define a new body plan within the pelagic durophagous stingrays.

**Conclusions:**

The significant morphological differences between *Promyliobatis* and extant representatives of Myliobatidae, Aetobatidae, Rhinopteridae, and Mobulidae, support its placement as separate stem group member. The phylogenetic placement of *Promyliobatis*, based on skeletal and dental characters, strongly supports its basal position within pelagic stingrays. However, its position within the Myliobatiformes becomes unstable when stingray taxa known by fossil teeth only are included. A comparative analysis of the skeletal and tooth morphologies, as well as of the evolutionary trends of pelagic stingrays is also discussed.

**Electronic supplementary material:**

The online version of this article (10.1186/s40851-019-0128-0) contains supplementary material, which is available to authorized users.

## Background

Pelagic stingrays are a group batoid fishes of the order Myliobatiformes characterized by a set of derived morphological characters (including wing-like pectoral fins with cephalic lobes anterior to the neurocranium and supported by the propterygia, anterior preorbital foramen located on the anterior aspect of nasal capsules, accessory hyomandibular cartilage, thickened jaws with fused antimeres and often supporting enlarged pavement-like dental plates formed by interlocking polyaulacorhizous teeth) which reflect the different swimming mode and feeding habits with respect to benthic stingrays [1–6]. Pelagic stingrays are mostly demersal to pelagic batoids. They are distributed worldwide, residing on continental and insular shelves, as well as in the open ocean, and feeding mainly on hard-shelled molluscs and crustaceans using their pavement-like plates [7, 8]. A reversal condition in the tooth morphology is present in devil rays, which possess small peg-like teeth that reflect their derived planktivorous feeding mode [9].

The pelagic stingrays include about 40 living species in five genera that are traditionally assigned to a single family, the Myliobatidae [3, 6, 8, 10]. However, according to the most recent classifications based on both molecular and morphological data, these taxa should be arranged in four families: the Myliobatidae (including seven species of the eagle ray *Aetomylaeus* Garman, 1908 [11], and 11 species of *Myliobatis* Cuvier, 1816 [12]), the Aetobatidae (five species of the pelagic eagle ray *Aetobatus* Blainville, 1816 [13]), the Rhinopteridae (eight species of the cownose ray *Rhinoptera* Cuvier, 1829 [14]), and the Mobulidae (eight species of the devil ray *Mobula* Rafinesque, 1810 [15]) [7]. White [16] synonymized the genus *Pteromylaeus* Garman, 1913 [17] with *Aetomylaeus* based on morphological and molecular evidences, whereas *Manta* Bancroft, 1829 [18] represents a junior synonym of *Mobula* according to White et al. [19].

The fossil record of the pelagic stingrays is remarkably rich, with about 150 nominal extinct species dating back to the latest Cretaceous and becoming more common and abundant in the Cenozoic [10, 20]. However, the fossil record is heavily biased towards isolated teeth, dermal denticles, and caudal spines, which are taxonomically and phylogenetically poorly informative, often leading to the attribution to the wastebasket genus *Myliobatis* [6, 21]. Only two taxa represented by nearly complete and articulated skeletal remains of pelagic stingrays have been recovered so far: *Weissobatis micklichi* Hovestadt and Hovestadt-Euler, 1999 [22] from the Oligocene Grube Unterfeld in Germany, and *Promyliobatis gazolai* (de Zigno, 1882) [23] from the Eocene Lagerstätte of Bolca in Italy [24, 25].

The celebrated Eocene (Ypresian, ca. 49 Ma [26]) Bolca Konservat-Lagerstätte from north-eastern Italy is one of the few Cenozoic deposits in which fossils of chondrichthyan fishes are exquisitely preserved and represented by nearly complete and articulated skeletons [25]. Along with bony fishes, they provide evidence of the recovery of shallow marine settings associated with reefs after the K-Pg extinction [27–29]. Ongoing studies are highlighting new insights into the palaeobiodiversity of chondrichthyans in this deposit, which includes possibly a dozen species-level taxa belonging to a variety of holocephalan, selachian, and batoid lineages, including chimaeriforms, carcharhiniforms, lamniforms, torpediniforms, rhinopristiforms and myliobatiforms [25, 30–34]. However, after the comprehensive account of cartilaginous fishes from Bolca published by Jaekel [35] no other systematic studies have been carried out on the pelagic stingrays to date. The aim of this paper is to re-describe the anatomy of the sole pelagic stingray taxon recovered from Bolca, *Promyliobatis gazolai*, in detail, also based on new material recently discovered in historical collections, and to discuss its relationships within the Myliobatiformes.

## Methods

This study is based on a re-examination of the holotypic specimen currently housed in the Museo Civico di Storia Naturale, Verona (MCSNV VII.B.90/91) and a second previously undescribed specimen that was discovered in the historical collection of the Museo di Storia Naturale of the Università degli Studi di Pavia (MSNPV 14620). Measurements were taken to the nearest 0.1 mm, and disc width (DW) is used throughout. Osteological and tooth terminologies mostly follow Nishida [1], Lovejoy [2], Carvalho et al. [3], and Hovestadt and Hovestadt-Euler [21].

The phylogenetic analysis is based on the morphological datasets of Marramà et al. [32, 36], which in turn are based on the matrix of Claeson et al. [10] extended with characters from Herman et al. [37–39], Schaefer and Summers [40], Aschliman et al. [5], and Last et al. [7, 41] [see Additional file 1]. The coding of characters 52, 54, 55, 64 and 65 for some taxa was updated following Blanco [42]. Moreover, ch. 9, describing the postorbital process of the neurocranium, has been changed to polymorphic (0/1) for *Aetomylaeus* following Aschliman [6]. Following the same author, ch. 16 (describing the fusion of jaw antimeres in pelagic stingrays) has been recoded as polymorphic for *Myliobatis*, since *M. freminvillei* exhibits the plesiomorphic condition by having the antimeres unfused [6]. Since the mesopterygium is absent (possibly fused to the scapulocoracoid) in *Aetomylaeus*, the state for ch. 27 has been changed from (1) to (2) following White [16], whereas it has been coded (1, fragmented) in *Weissobatis* following the reconstruction in Hovestadt and Hovestadt-Euler [22]. The presence of a greatly elongated median prepelvic process of the puboischiadic bar, typical of freshwater potamotrygonids, has been reported also for *Rhinoptera* and *Mobula* [6] and their state (ch. 30) has been consequently recoded. Because of the extremely high ontogenetic, inter- and intraspecific variation in tooth morphologies in *Myliobatis* and *Aetomylaeus* [21] the coding for chs. 50 to 52 cannot be restricted to one single state for these taxa. Therefore, we prefer to consider their condition to be polymorphic, as well as that of *Myliobatis* described in ch. 63. Moreover, following the description of Claeson et al. [10] the state of ch. 50 for *Rhinoptera* has been changed from (1) to (0).

We performed two different phylogenetic analyses to test the quality of data: in the first one we included only fossil stingray taxa based on holomorphic specimens (i.e. articulated skeletal material). In the second analysis we also included those species known only by isolated teeth or dental plates, present in Claeson et al. [10]. For this latter analysis we used the original statements of Claeson et al. [10] since some of these fossil taxa show states that are not present in recent or holomorphic fossil taxa.

The matrix was compiled in MESQUITE v.3.03 [43] and the phylogenetic analysis was performed with TNT v.1.5 [44]. Following Claeson et al. [10], we used the branch-and-bound method with 1000 replicates of random stepwise addition (branch swapping: tree-bisection-reconnection) and holding one tree at each step. All the characters are unordered and given equal weight.

### Institutional abbreviations

EMRG, Evolutionary Morphology Group, Department of Palaeontology of University of Vienna; MCSNV, Museo Civico di Storia Naturale di Verona; MSNP, Museo di Storia Naturale dell’Università degli Studi di Pavia.

## Results

### Systematic palaeontology

Chondrichthyes Huxley, 1880 [45]

Batomorphii Cappetta, 1980 [46]

Myliobatiformes Compagno, 1973 [47]

Myliobatoidea Compagno, 1973 [47]

*Promyliobatis* Jaekel, 1894 [35]

#### Type species

*Myliobates gazolai* de Zigno, 1882 [23]

#### Diagnosis

A pelagic stingray unique in having the following characters: tail sting origin displaced posteriorly on the tail, at about 50–60% of tail length (vs. proximally on the tail and just posterior to the pelvic fins in other pelagic stingrays), pectoral fins joining in front of the head (vs. join the head laterally in other pelagic stingrays), anterior and posterior pectoral-fin margins nearly straight (vs. concave or convex in other pelagic stingrays), compagibus laminam absent (vs. present or poorly developed in other pelagic stingrays), mesopterygium as a single element (vs. fragmented or fused to scapulocoracoid in other pelagic stingrays). Moreover, *Promyliobatis* is characterized by a combination of plesiomorphic traits, including: anterior margin of cephalic lobes continuous (vs. single with an indentation in the Aetobatidae, and completely separated in two distinct cephalic fins in both the Rhinopteridae and Mobulidae); continuity of pectoral-rostral radials (vs. interrupted in all the other genera, except in *Myliobatis*); rostral radials less developed than pectoral radials (vs. equally developed in *Myliobatis*); pelvic girdle almost straight or slightly bent (vs. strongly bent in the Aetobatidae, Rhinopteridae and Mobulidae); median prepelvic process absent (vs. present in the Rhinopteridae and Mobulidae); crushing/grinding pavement-like dentition formed by interlocked expanded teeth (vs. small individual peg-like teeth in the Mobulidae); about 218 vertebrae (of which 20–22 are monospondylous and 148 are diplospondylous anterior to the sting, and 50 diplospondylous posterior to the sting); about 87 pectoral radials (excluding rostrals) of which 35 are propterygial, 10–12 mesopterygial, and 40 metapterygial; 22 or 23 pelvic radials; one row of hexagonal and mesio-distally enlarged symphyseal teeth (width/length ratio 3.6–4.5), two rows of hexagonal or rhomboidal lateral teeth, and a single row of posterior teeth in both the upper and lower plates.

#### Included species

Type species only, by monotypy.

#### Remarks

**Fig. 1:**
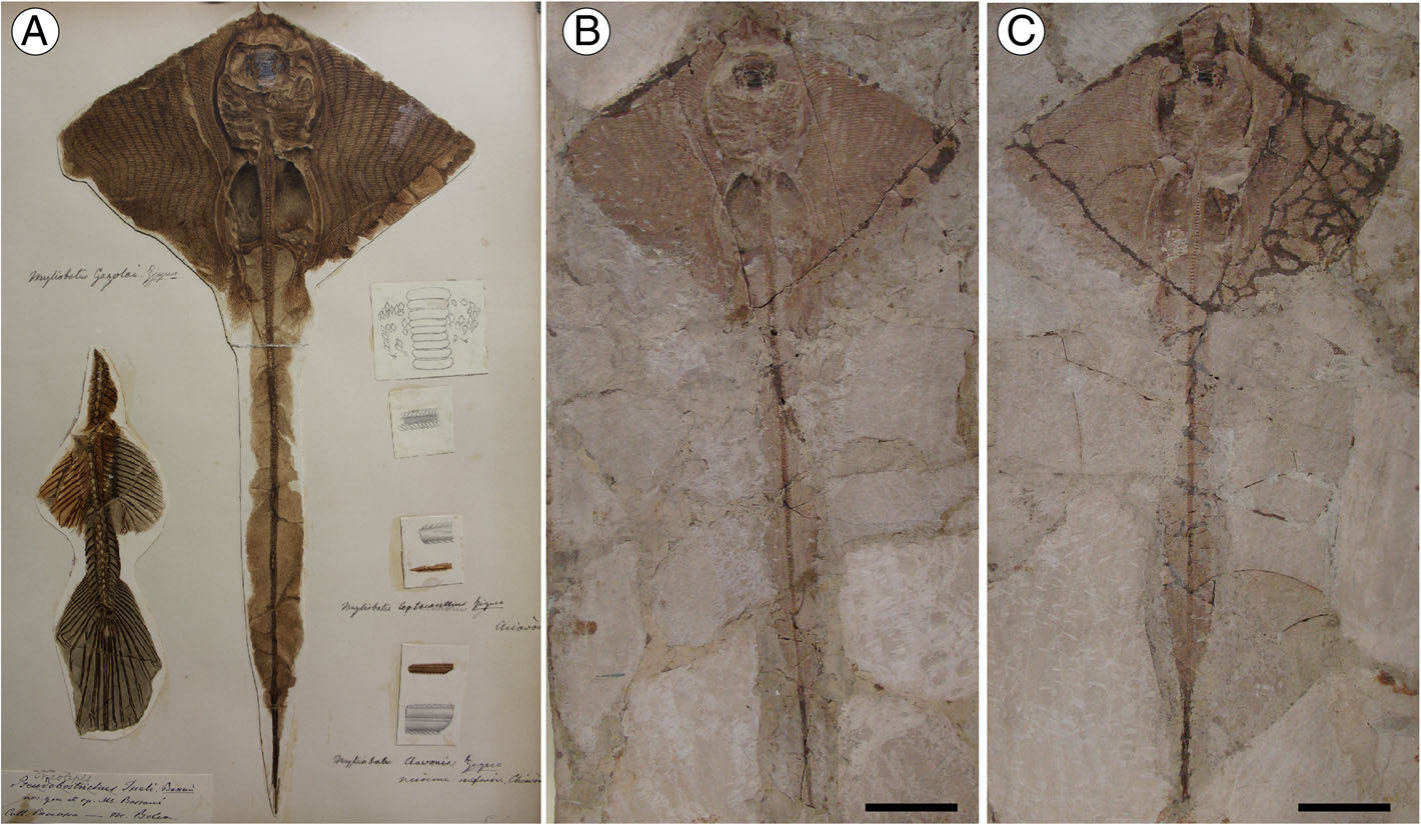
*Promyliobatis gazolai* from the Eocene of the Bolca Lagerstätte. **a** Original historical plate of the holotype, illustrated and specified as *Myliobates gazolai* in de Zigno [23] and in de Zigno [49]; photo: courtesy of Università degli Studi di Padova. **b**, **c** MCSNV VII.B.90/91, holotype, in part and counterpart. Scale bars = 50 mm

The first report of an articulated pelagic stingray from Bolca Lagerstätte was provided by de Zigno [23] who, examining a single specimen in part and counterpart (MCSNV VII.B.90/91) from the Gazola collection in Verona, recognized its affinities with the modern eagle rays and created the species *Myliobates gazolai*, providing a description and a remarkably detailed drawing (Fig. 1a). Afterward, in his comprehensive review of the Bolca chondrichthyans, Jaekel [35] undertook a re-examination of the holotype, and highlighting some morphological differences with the living *Myliobatis*, created the new genus *Promyliobatis*. Although the affinities of *Promyliobatis* with pelagic durophagous stingrays are clear, a detailed morphological analysis with an associated hypothesis about its relationships within living and extinct myliobatiforms remains elusive. Hovestadt and Hovestadt-Euler [22] and Carvalho et al. [3] clearly pointed out the unquestionable alignment of *Promyliobatis* within the group of pelagic stingrays, and provided a tentative phylogenetic hypothesis that placed this extinct genus near the base of the clade of pelagic stingrays. Although several authors agreed with the separate taxonomic status of *Promyliobatis* from other pelagic stingrays (see synonymy), Cappetta [20, 48] considered the genus a junior synonym of *Myliobatis*, possibly due to the difficulty to recognize unambiguous tooth characters to separate the two genera. However, although the dental plates are very similar, our detailed re-examination of the skeletal anatomy highlights important differences between *Promyliobatis*, *Myliobatis* and any other pelagic stingray, corroborating its separate taxonomic status.

*Promyliobatis gazolai* (de Zigno, 1882) [23]

1882. *Myliobates gazolai*; de Zigno [23], p. 682, pl. 5, Figs. 1, 2, 3 (first occurrence of name, description and reconstruction)

1885. *Myliobates gazolai*; de Zigno [49], p. 7, Figs. 1, 2, 3

1894. *Promyliobatis gazolae*; Jaekel [35], p. 152, Fig. 32, pl. 6

1904. *Promyliobatis gazolae*; Eastman [50], p. 27

1905. *Promyliobatis gazolae*; Eastman [51], p. 352

1906. *Promyliobatis gazolai*; Leriche [52], p. 378

1922. *Promyliobatis gazolai*; D’Erasmo [53], p. 13

1987. *Myliobatis gazolai*; Cappetta [48], p. 172

1991. *Promyliobatis gazolae*; Frickhinger [54], p. 215

1999. *Promyliobatis gazolae*; Hovestadt and Hovestadt-Euler [22], p. 337, Fig. 4c

2004. *Promyliobatis gazolae*; Carvalho et al. [3], p. 9, Figs. 49A, 50

2012. *Myliobatis gazolai*; Cappetta [20], p. 451

2013. *Myliobates gazolai*; Hovestadt and Hovestadt-Euler [21], p. 39, pl. 32, Fig. 1

2014. *Promyliobatis gazolae*; Carnevale et al. [55], p. 41

2018. *Promyliobatis gazolae*; Marramà et al. [25], p. 287, Fig. 10

#### Holotype

MCSNV VII.B.90/91, nearly complete and well-preserved articulated skeleton, lacking the distal-most portion of the tail and showing almost complete upper and lower tooth plates, in part and counterpart; 233.3 mm DW (Fig. 1).

#### Referred material

**Fig. 2:**
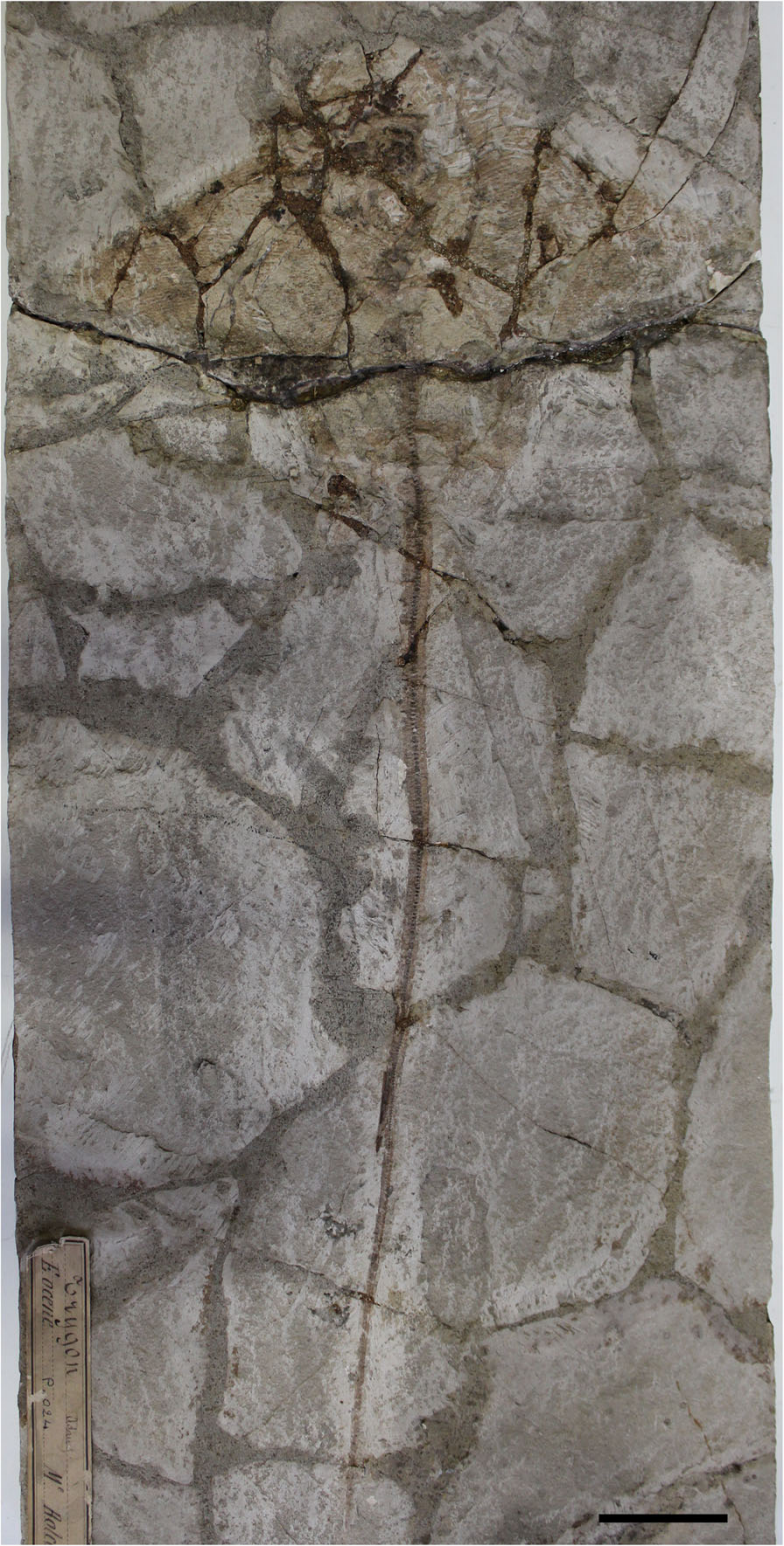
*Promyliobatis gazolai* from the Eocene of the Bolca Lagerstätte. MSNPV 14620. Scale bar = 50 mm

MSNPV 14620, partially complete articulated skeleton showing partially complete upper and lower tooth plates; 225.8 mm DW (Fig. 2).

#### Occurrence

Pesciara site, Bolca Konservat-Lagerstätte, Italy; early Eocene, late Ypresian, middle Cuisian, SBZ 11, *Alveolina dainelli* Zone [see Additional file 1 for information on geological setting].

#### Diagnosis

As for the genus.

### Description

#### General morphology

*Promyliobatis gazolai* is represented by two partially complete and articulated skeletons. The holotype (MCSNV VII.B.90/91) apparently lacks part of the tail, which instead is complete in the second specimen (MSNPV 14620) (Figs. 1 and 2). Due to the overall good preservation it was possible to recognize and describe several skeletal characters, which allow comparing the skeletal anatomy of *Promyliobatis* with that of other known pelagic stingrays. Measurements and meristics of *P. gazolai* are given in Table 1. The two specimens are similar in size suggesting similar ontogenetic stages, with the largest one (the holotype) being 233 mm in DW and possibly 50–60 cm in total length. The pectoral disc of *Promyliobatis gazolai* is rhomboid, wing-like, each fin being triangular, with anterior and posterior edges having the same length and with angular or pointed lateral apices. The pectoral disc is broader than long (disc length about 60–70% of DW), whereas the total length is about 220% of DW in the most complete specimen. As noticed by Hovestadt and Hovestadt-Euler [22], *Promyliobatis* is unique among eagle rays since its pectoral fins join each other in front of the head, whereas in *Weissobatis*, *Aetomylaeus*, *Myliobatis* and *Aetobatus* the pectoral fins join the head laterally. The tail is long, about 166% of DW.

**Table 1:** Morphometric and meristic data of the two specimens of *Promyliobatis gazolai*

	MCSNV VII.B.90/91		MSNPV 14620	
Measurements	mm	% DW	mm	% DW
Total length	?	?	497.3	220.2
Disc length	164.0	70.3	134.9	60.8
Disc width	233.3	100.0	225.8	100.0
Tail length	?	?	375.6	166.3
Preoral length	23.1	9.9	?	?
Mouth-scapulocoracoid distance	64.5	27.7	69.5	30.8
Scapulocoracoid width	48.5	20.8	46.1	20.4
Pelvic girdle width	40.2	17.2	42.9	19.0
Sting length	50.8	21.8	51.5	22.8
Pelvis-tip of tail length	?	?	333.2	147.6
Pre-sting length	393.9^a^	168.8 ^a^	319.9	141.7
Distance from tip of disc to max width disc	82.8	35.5	62.5	27.7
Prepelvic distance	136.6	58.6	130.1	57.6
Prescapular distance (head length)	64.8	27.8	80.4	35.6
Pelvic fin length	47.6	20.4	?	?
Pectoral-fin insertion to sting	240.6 ^a^	103.1 ^a^	181.3	80.3
Meristics				
Propterygial radials	35		?	
Mesopterygial radials	12		10	
Metapterygial radials	40		?	
Total pectoral radials	87		?	
Pelvic radials	22–23		?	
Vertebrae from scapulocoracoid to pelvic girdle	22		20	
Vertebrae from pelvic girdle to sting	?		148	
Vertebrae posterior to sting	?		50	
Total vertebrae	?		218	
Sting serrations per side	?		25	

^a^ Data might not be reliable because the tail sting of MCSNV VII.B.90/91 appears to have been positioned incorrectly during the historical restoration of the slab

#### Neurocranium

**Fig. 3:**
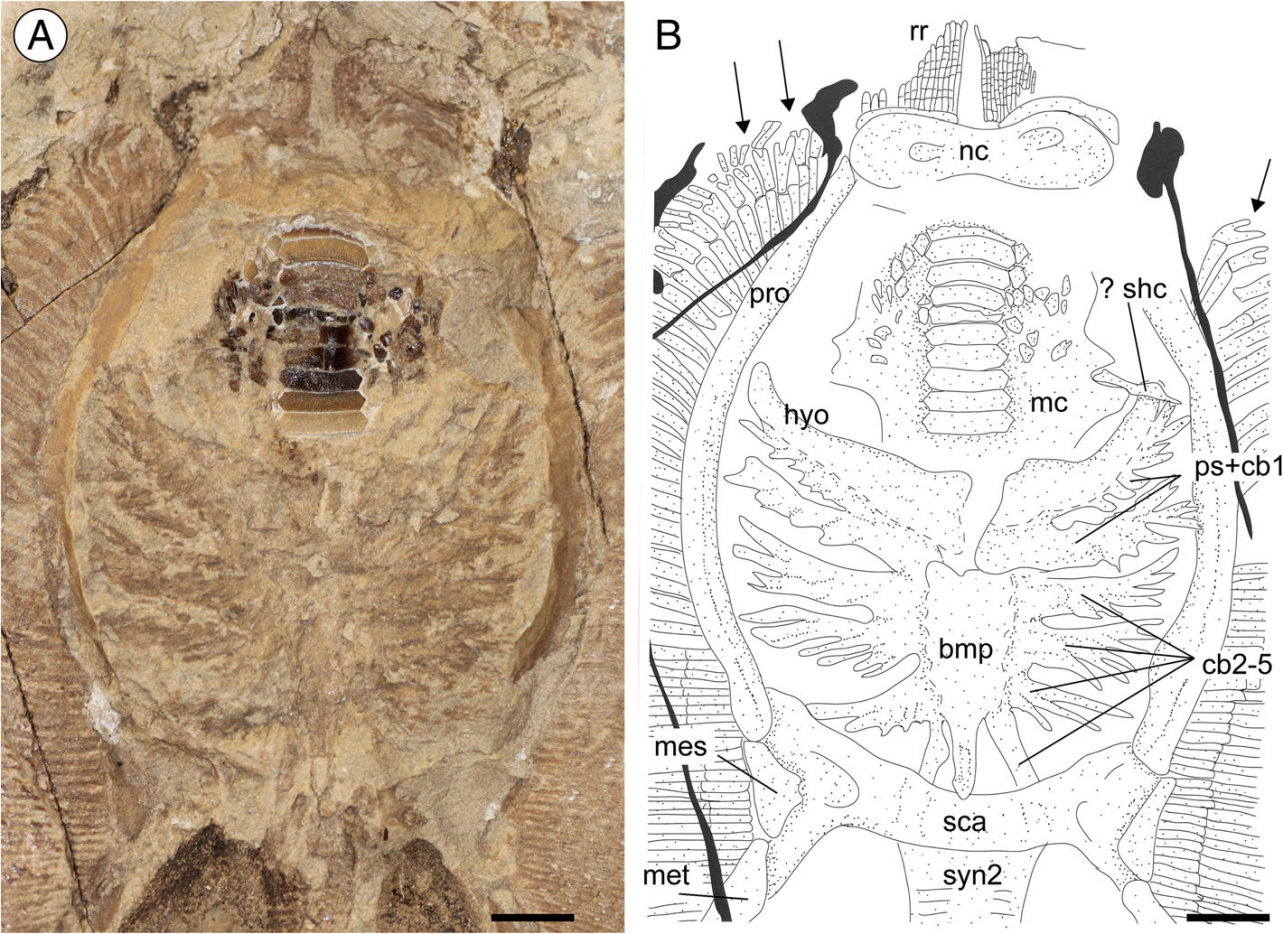
*Promyliobatis gazolai* from the Eocene of the Bolca Lagerstätte. **a** Detail of the head region of the holotype MCSNV VII.B.90/91. **b** Reconstruction. Scale bars = 10 mm. Abbreviations: bmp, basibranchial medial plate; cb, ceratobranchials; hyo, hyomandibula; mc, Meckel’s cartilage; mes, mesopterygium; met, metapterygium; nc, nasal capsules; pro, propterygium; ps, pseudohyoid; rr, rostral radials; sca, scapulocoracoid; shc, secondary hyomandibular cartilage; syn2, thoracolumbar synarcual. The arrows indicate the anterior-most propterygial radials, which are branched and do not present interradial joints, therefore excluding the presence of the compagibus laminam

The whole neurocranium is poorly preserved (Fig. 3). The rostral cartilage is clearly absent as in all stingrays [56] and, anterior to the nasal capsules, the neurocranium lacks the anterior processes typical of *Rhinoptera* and *Mobula* [3]. The nasal capsules are possibly ventro-laterally expanded, ovoid in shape, wider than long and with a centrally concave anterior margin.

#### Jaws, hyoid and gill arches

Both the palatoquadrate and Meckel’s cartilage are largely incomplete although the outline of the latter can be recognized around the lower tooth plate in MCSNV VII.B.90/91 (Fig. 3). The hyomandibulae are long, slender distal to the articulation with the Meckel’s cartilage, and more stout and robust proximally, where they articulate with the occipital region of neurocranium. A small and thin cartilaginous element seems to connect the distal tip of the hyomandibula to the Meckel’s cartilage, possibly representing the secondary hyomandibular cartilage characteristic of *Urolophus* and pelagic stingrays [2, 3]. The ventral gill arches of *Promyliobatis* appear to be partially preserved in the holotype and their morphology is consistent, at least in part, with that of pelagic stingrays as reported by Miyake and McEachran [57]. The central medial plate, resulting from the fusion of the basibranchial copula and the basibranchial components, is tubular and slightly compressed laterally with the posterior distal tip tapering into a small median projection. The anterior margin of the basibranchial medial plate appears to be straight without the anterior median projection that is most characteristic for benthic stingrays [3, 58]. The absence of the basihyal appears to be genuine, being absent in all the pelagic stingrays [3, 57]. There are five pairs of ceratobranchials, articulated with the lateral margins of the basibranchial medial plate, hidden by the associated filamentous branchial filaments. Although partially hidden by the hyomandibula, the first pair of ceratobranchials appear fused proximally to the pseudohyoid. The fourth and fifth ceratobranchials are possibly fused to each other.

#### Synarcuals and vertebral column

**Fig. 4:**
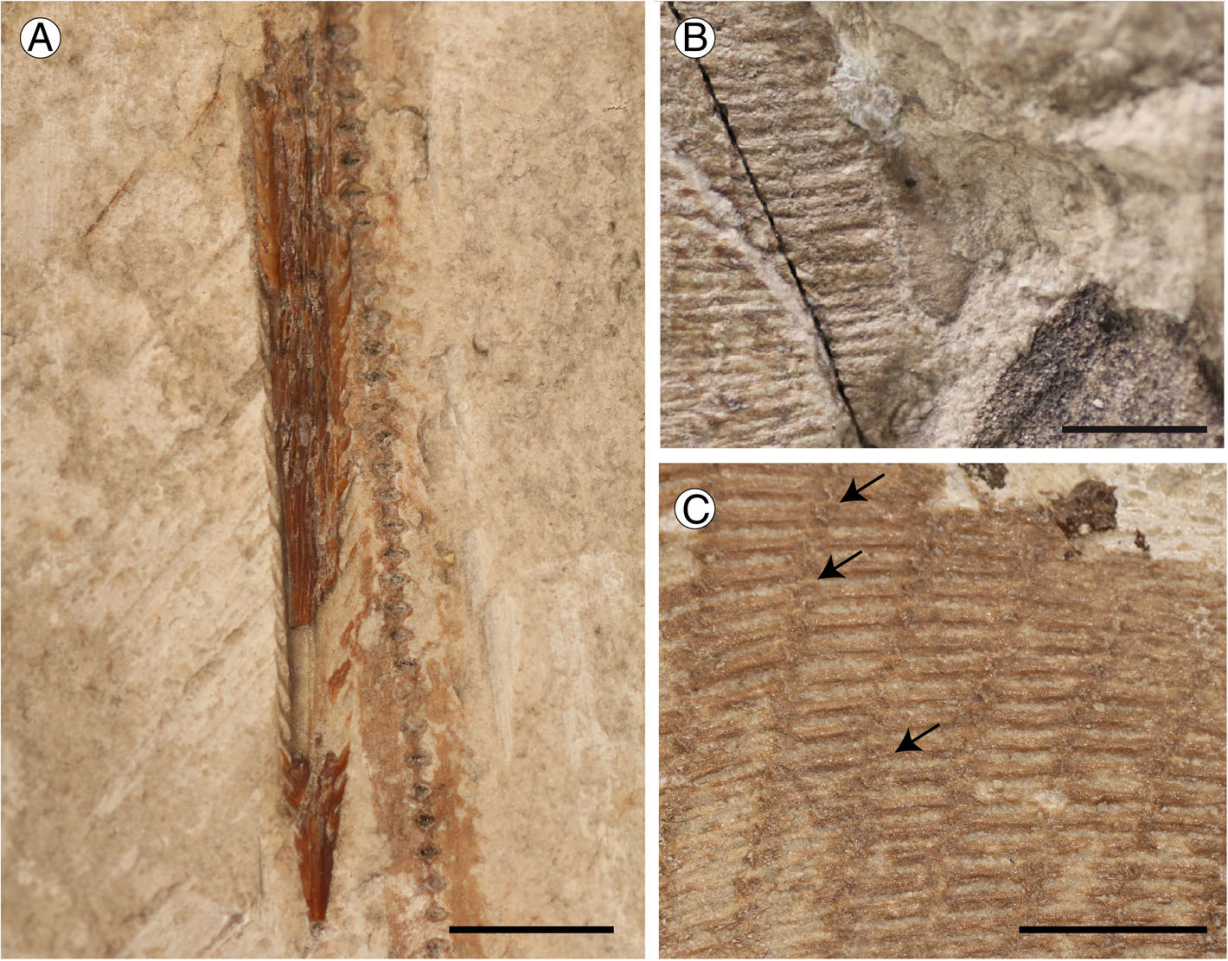
*Promyliobatis gazolai* from the Eocene of the Bolca Lagerstätte. **a** Detail of the tail sting of MSNPV 14620. **b** Close up of the single and non-fragmented mesopterygium in MCSNV VII.B.90/91. **c** Detail of the pectoral radials in MCSNV VII.B.90/91; note the crustal calcification of radials and the interradial joints indicated by arrows. Scale bars = 10 mm

The morphology of the first (cervicothoracic) synarcual is difficult to determine since it is largely hidden under the gill skeleton. The second (thoracolumbar) synarcual articulates anteriorly with the cervicothoracic synarcual; it is triangular and tapers posteriorly, reaching the mid-length between the pectoral and pelvic girdles. About ten unfused vertebral centra can be recognized throughout the thoracolumbar synarcual length. The vertebral column of *Promyliobatis gazolai* consists of about 218 vertebral centra (counted in MSNPV 14620). There are 20–22 trunk (monospondylous) centra from the first distinguishable centrum to the anterior margin of the puboischiadic bar. About 148 diplospondylous centra can be recognized from the anterior margin of the puboischiadic bar to the sting origin, and about 50 are present between the sting origin and the cartilaginous tail rod. The vertebral centra are very small, subrectangular in shape with an almost similar length and width. Small neural spines are visible in MSNPV 14620 from the proximal region of the tail up to just posterior the sting (Fig. 4 a). The distal portion of the vertebral column is stiffened by the presence of a cartilaginous rod, which is typically present in dasyatids, potamotrygonids and pelagic stingrays [3]. Ribs are absent.

#### Pectoral fins and girdle

The scapulocoracoid of *Promyliobatis* is almost straight and robust measuring about 20% of DW, being located ventrally to the cervicothoracic synarcual. However, it is difficult to distinguish the morphology of the scapular processes and the coracoid bar in the examined material, as well as the fusion between the suprascapulae with the median crest of the cervicothoracic synarcual. Laterally, the scapulocoracoid bar articulates with the pterygia. The propterygium is quite long and arched, and tapers distally extending to the anterior disc margin. The propterygium is distally segmented with the first segment adjacent to the anterior margin of the nasal capsules resembling the condition observed in the pelagic stingrays [5]. The proximal portion of the propterygium is large, and articulates with the anterior portion of the lateral margin of the scapulocoracoid and possibly with the mesopterygium. As in all other pelagic stingrays, *Promyliobatis* possesses cephalic lobes supported by the anterior-most radials (about 12–15 per side) of the first propterygial segment, which are located anteriorly to the nasal capsules. The anterior margin of cephalic lobes appears to be continuous, similar to the condition of *Myliobatis* and *Aetomylaeus*, and different from that of *Aetobatus* (single with an indentation), *Rhinoptera* and *Mobula* (completely separated in two distinct cephalic fins) [3]. It is unclear whether the rostral lobes are connected to the pectoral disc by a subocular ridge like in *Myliobatis* [16] or not, but as noticed by Jaekel [35] the pectoral radials appear to be continuous with the rostral lobe (Fig. 3), thereby resembling the condition that is typical for *Myliobatis* [16]. Although the rostral radials were considered absent in *Weissobatis* by Hovestadt and Hovestadt-Euler [22], it is likely that the condition is due to the poor preservation of this region in the two specimens examined. The rostral radials of *Promyliobatis* are thin and much less developed than the pectoral radials, similar to those of *Aetobatus* and *Aetomylaeus* [16]. A single and non-fragmented mesopterygium is present, characterized by an external margin that is nearly straight, and not fused to radials (Figs. 3 and 4 b). The mesopterygium of *Promyliobatis* has a unique configuration being completely different from those observed in the other families of pelagic stingrays. The mesopterygium of *Myliobatis* is fragmented, whereas it is missing/fused to scapulocoracoid in *Aetomylaeus, Aetobatus*, *Rhinoptera* and *Mobula* [3, 16]. Its status was not described in *Weissobatis* although it was figured as fragmented [22]. The metapterygium is slightly longer than the propterygium, arched and tapers distally, ending slightly posteriorly to the anterior margin of the puboischiadic bar. There are about 87 pectoral radials of which 35 are propterygial (rostrals excluded), 10–12 are mesopterygial, and about 40 are metapterygial. The distribution of the pectoral radials of *Promyliobatis* is consistent with that of the oscillatory swimmers in which the number of metapterygial radials is higher than that of the propterygial radials [59]. Each radial is composed of at least 15 segments. The radials (Fig. 4 c) are highly calcified, forming the so-called ‘crustal’ calcification typical of batoids with oscillatory swimming mode, including *Gymnura* and all the pelagic stingrays [40]; the radials also show the lateral expansions that articulate with the surface of the adjacent radials. Moreover, *Promyliobatis* does not exhibit the compagibus laminam. This derived structure is found only in the anterior portion of the pectoral fins of *Aetomylaeus*, *Aetobatus*, *Rhinoptera*, and *Mobula* and consists of a set of condensed propterygial radials that have interradial fin ray joints and no terminal branching [59]. *Myliobatis* exhibits a variety of conditions concerning the compagibus laminam. For example, *M. hamlyni* has a compagibus laminam, whereas this feature is lacking in *M. californica* and *M. goodei*, and *M. freminvillei* shows an intermediate morphology [59]. As in *M. goodei* and *M. californica*, the anteriormost propterygial radials of *Promyliobatis gazolai* are branched and without interradial joints (Fig. 3) thereby exhibiting the plesiomorphic condition for stingrays.

#### Pelvic girdle and fins

**Fig. 5:**
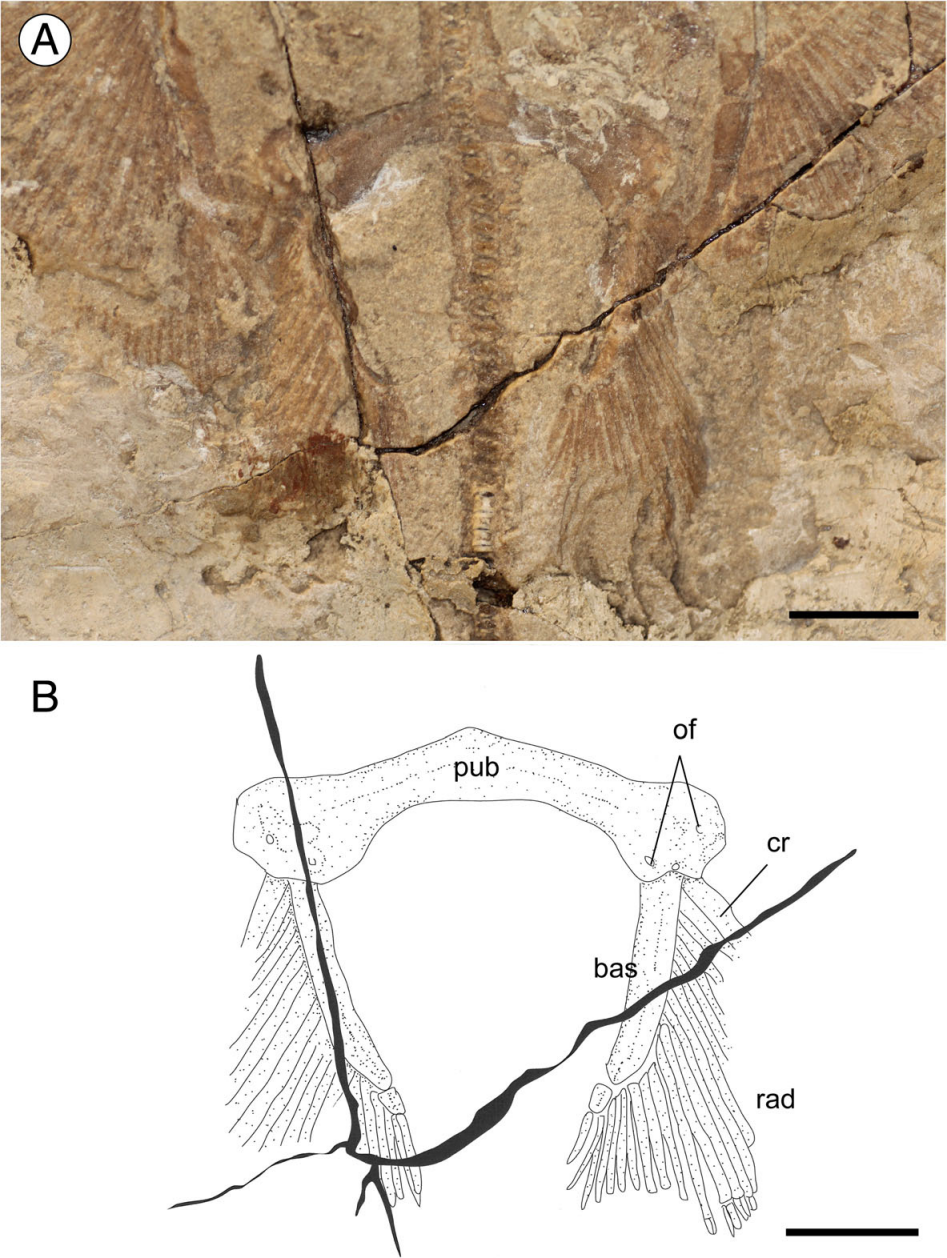
*Promyliobatis gazolai* from the Eocene of the Bolca Lagerstätte. **a** Detail of the pelvic region of the holotype MCSNV VII.B.90/91. **b** Reconstruction. Abbreviations: bas; basipterygium; cr, compound radial; of, obturator foramina; pub, puboischiadic bar; rad, pelvic radials. Scale bars = 10 mm

The pelvic fins of *Promyliobatis* are single-lobed, protruding beyond the pectoral disc for about half of their length, which equals about 20% of DW. The puboischiadic bar (Fig. 5) is robust, relatively wide (about 23% of DW), and only moderately arched, contrary to the condition that is characteristic for the Aetobatidae, Rhinopteridae and Mobulidae in which it is considerably arched [3, 16]. The bar is enlarged at its distal edges where there are two or three obturator foramina. The long median prepelvic process typical of freshwater potamotrygonids, *Rhinoptera* and *Mobula* [6] is clearly absent. The iliac processes are not preserved in both the examined specimens. The basipterygia are slightly shorter than the puboischiadic bar width, and are approximately straight or with a slightly concave inner margin. Each basipterygium supports 22 or 23 pelvic rays, including the first compound radial, which bifurcate distally. There is no evidence of the claspers, suggesting that both the specimens represent female individuals.

#### Dorsal and caudal fin

A single dorsal fin is usually present just anterior to the tail sting in all the pelagic stingrays [3]. However, due to the poor preservation of this region, it is unclear whether it is present in *Promyliobatis* or not, although it very likely was. A complete caudal fin is clearly absent. Jaekel [35] reported the presence of long tail folds in the holotype MCSNV VII.B.90/91. However, our analysis of the two specimens did not recognize dorsal or ventral elements (rudimentary radials of Nishida [1]) supporting dorsal and ventral folds; the report of these structures by Jaekel [35] may have been biased by the different colouration of the sedimentary matrix of the fossil and that of the small pieces traditionally used to assemble the slab (see also Fig. 1). Within the stingrays, tail folds are usually present in some dasyatids and potamotrygonids [3]. Although the tail folds have been considered absent in *Gymnura* and in all the pelagic stingrays, their presence recently has been reported in some species of *Gymnura*, *Myliobatis* and *Aetomylaeus* [60–62]. However, their homology with the folds typical of benthic stingrays cannot be determined since it is unclear if radials support the folds of these genera. In our opinion, it is most parsimonious to consider the cartilaginous elements observable in the tail of specimen MSNPV 14620 (Fig. 4 a), as neural spines, rather than radial elements supporting the tail folds, which are absent in pelagic stingrays.

#### Dentition

**Fig. 6:**
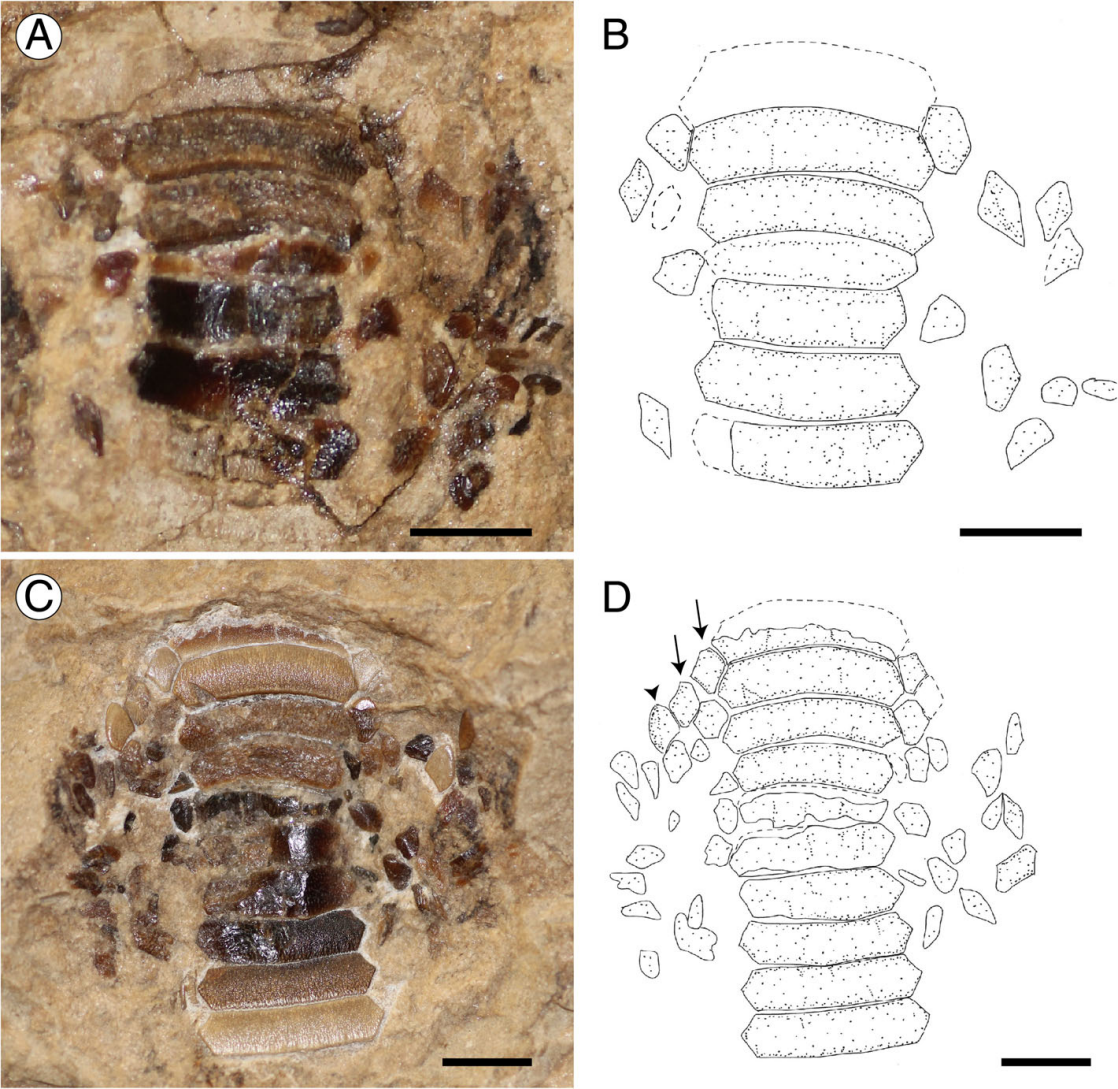
Dentition of *Promyliobatis gazolai* from the Eocene of the Bolca Lagerstätte; specimen MCSNV VII.B.90/91. **a** Upper tooth plate. **b** Reconstruction. **c** Lower tooth plate. **d** Reconstruction. Scale bars = 5 mm. The arrows indicate the two rows of lateral teeth on lower tooth plate; arrowhead indicates the posterior row. Rostral direction upward

**Fig. 7:**
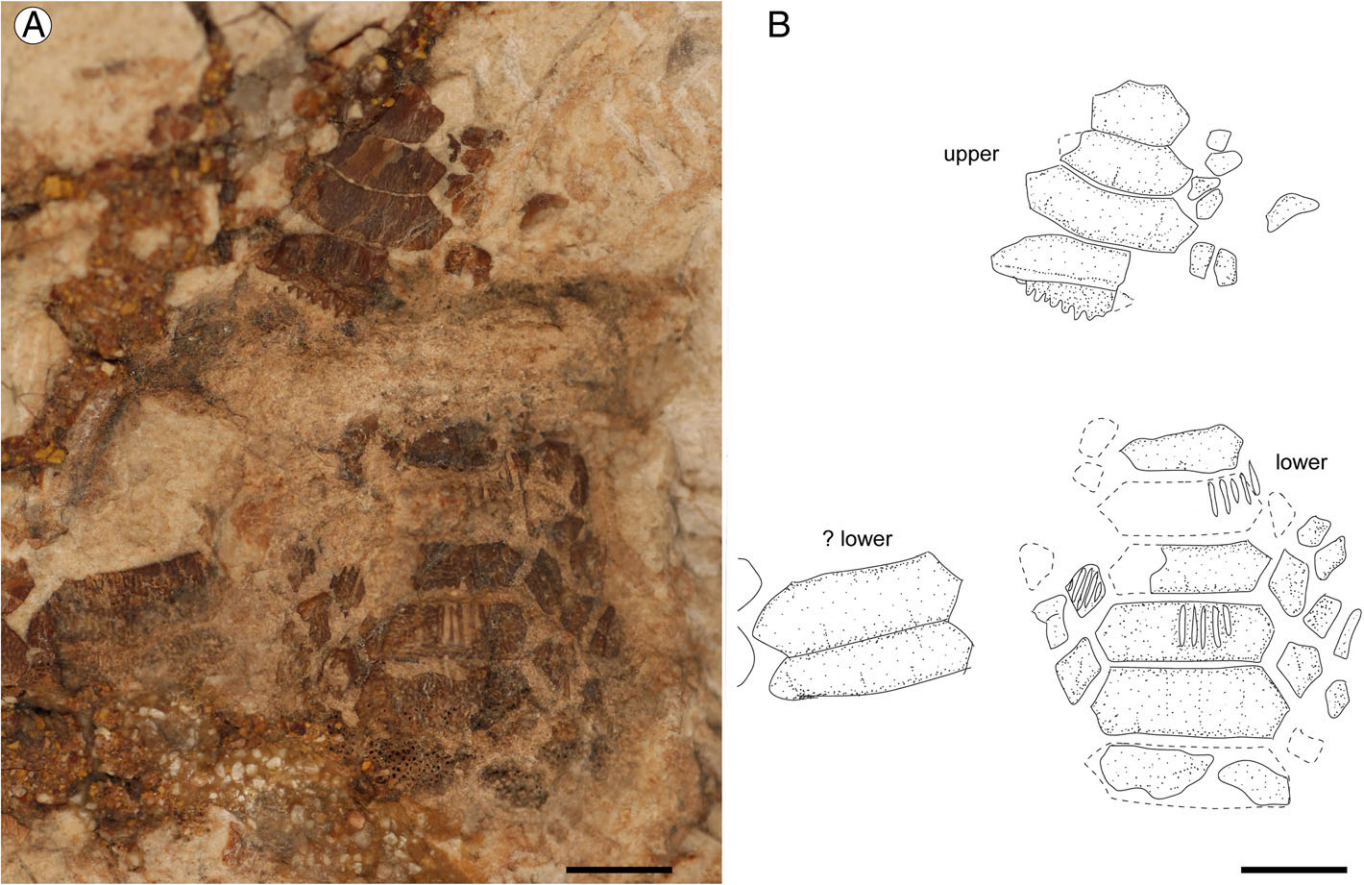
Dentition of *Promyliobatis gazolai* from the Eocene of the Bolca Lagerstätte; specimen MSNPV 14620. **a** Upper and lower tooth plates. **b** Reconstruction. Scale bars = 5 mm

*Promyliobatis* exhibits the typical grinding-type dentition of durophagous pelagic stingrays with broad teeth in pavement-like arrangement (Figs. 6, 7), very similar to those of *Myliobatis* [21, 39]. The nearly complete dentition of MCSNV VII.B.90/91 shows that teeth of *P. gazolai* are arranged in at least seven rows in both upper and lower jaws, of which one symphyseal, two laterals and one posterior per side (Fig. 6). The occlusal surfaces of both the upper and lower tooth plates appear smooth, straight and without ornamentation. It is not possible to observe the morphology of the tooth interlocking mechanism, although it must have been tightly since the teeth were found mostly associated.

In the lower tooth plate, there are up to ten symphyseal teeth, which are hexagonal in shape and laterally expanded, being from 3.8 (in MSNPV 14620) to 4.5 times (in MCSNV VII.B.90/91) wider than long. Their lingual and labial margins are slightly bent in MCSNV VII.B.90/91, although they appear nearly straight in MSNPV 14620. The lateral and posterior teeth of the lower jaw are mostly scattered in both specimens, but in MCSNV VII.B.90/91 it is possible to recognize at least two lateral and a single posterior row (Fig. 6 c-d). Lateral teeth are mostly hexagonal to rhomboid in shape. The posterior teeth have mostly a triangular shape with a long, labio-lingually directed, and nearly straight distal edges.

There are up to seven symphyseal teeth in the upper tooth plate, which appear slightly bent, laterally expanded and about four times wider than long. The number and morphology of the lateral and posterior teeth in upper tooth plates are similar to those of the lower plate.

The root is clearly polyaulacorhizous, with grooves regularly spaced and wider than the root laminae. It was not possible to examine the pulp cavity and the tooth vascularization, but it is very likely that they are similar to those of living eagle rays.

A certain degree of variation can be observed in the dentition of the two specimens, mostly in the proportions and number of symphyseal teeth. However, due to the extremely high intraspecific variation of eagle ray dentitions [21], we interpret these differences as related to individual variability. The rapid increase in tooth size in the upper symphyseal teeth of MSNPV 14620 (Fig. 7) might suggest that the individual was a young adult or even juvenile.

#### Squamation and sting

Like all the extant pelagic stingrays, the two specimens of *Promyliobatis* lack dermal denticles and thorns. A single serrated caudal sting with a length of about 22–23% of DW is present in both the available specimens (Fig. 4 a). The sting is elongate, dorso-ventrally flattened and tapers toward the apex. In extant stingrays the caudal stings are usually set proximally on the tail and are placed just posterior to the pelvic fins in pelagic stingrays. In *P. gazolai* the caudal sting is placed farther posteriorly on the tail, at about its mid-length in MSNPV 14620. Its location near the tip of tail in the holotype MCSNV VII.B.90/91 is possibly due to erroneous historical reconstruction or, more likely, to the lack of the distal-most part of the tail. About 25 small, oblique and hook shaped serrations per side are present in the sting of MSNPV 14620. There are no particular diagnostic features that are useful to separate the sting of *Promyliobatis* from those of other eagle rays, since the characters of the stings of myliobatids are mostly uninformative from a taxonomic point of view [21].

### Phylogenetic analysis

**Fig. 8:**
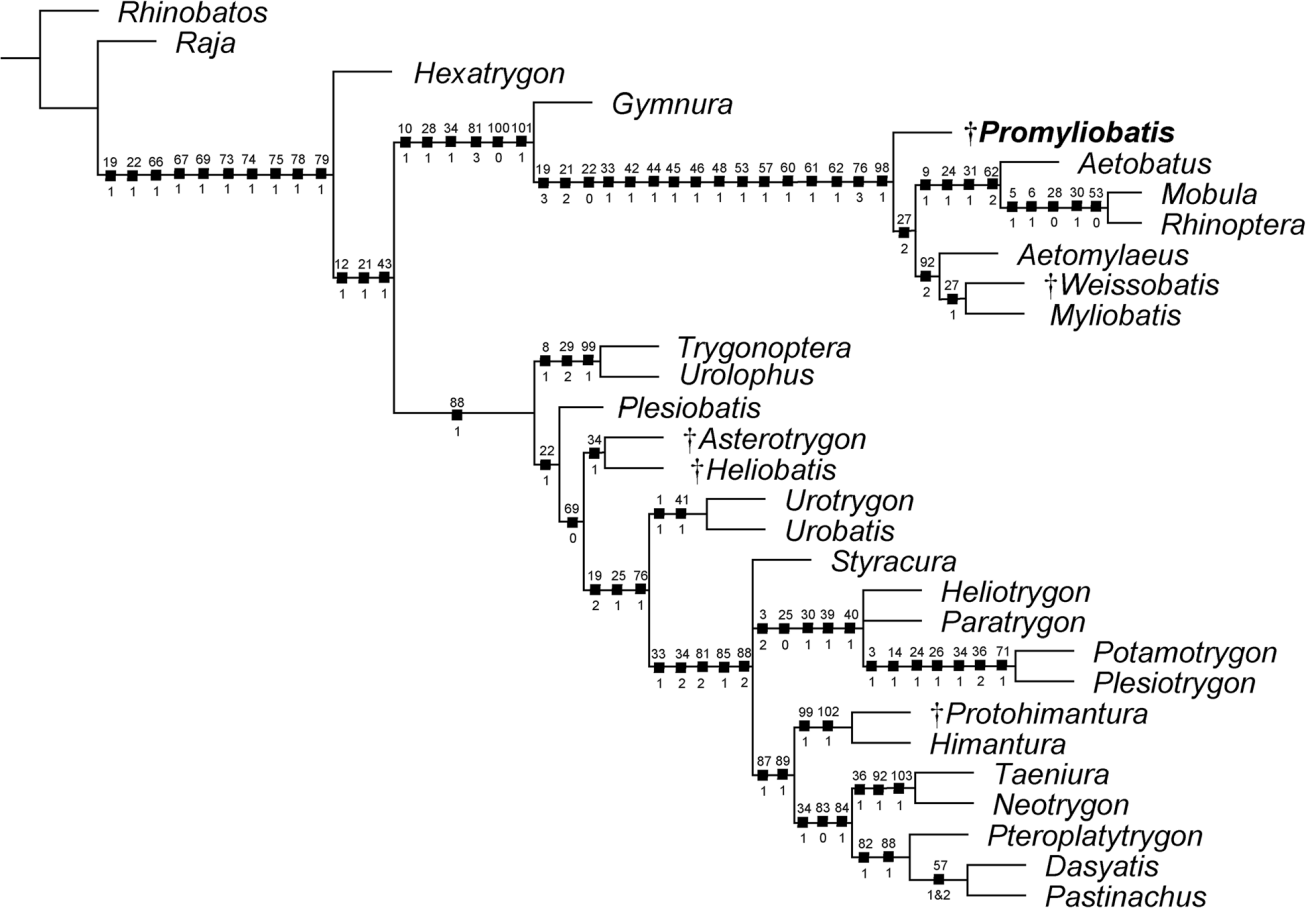
The single tree retrieved by the branch-and-bound method showing the hypothetical relationships of *Promyliobatis gazolai* within the Myliobatiformes using living and fossil taxa based on holomorphic specimens. Number character above and state below on each node. Extinct taxa are marked with a dagger preceding their name

In their account on the Eocene freshwater stingrays from Green River Formation, Carvalho et al. [3] proposed a phylogenetic hypothesis that tentatively placed *Promyliobatis* close to the extant *Myliobatis*. A tentative phylogenetic analysis was also provided by Hovestadt and Hovestadt-Euler [22] who placed *Promyliobatis* as the basalmost eagle ray (excluding *Pteromylaeus*), and *Weissobatis* as an intermediate form between *Promyliobatis* and a sister group formed by *Aetomylaeus* and *Myliobatis*. However, the authors did not indicate which traits characterize the more derived condition of all the eagle rays with respect to the *Pteromylaeus* complex, and the use of some ambiguous characters (e.g. presence/absence of the caudal sting, thickness of the pterygia) may have led to a tree topology different from that of the present study. Our analysis of 103 traits coded for 30 taxa with the branch-and-bound method produced a single parsimonious tree of 211 steps, a CI 0.65, and a RI 0.81 that resolved the systematic affinities of *Promyliobatis* (Fig. 8). The tree recovered is similar to the ones depicted by Marramà et al. [32, 36] including an improved resolution of the positions of *Plesiobatis* as well as of the Eocene freshwater stingrays *Asterotrygon* and *Heliobatis*. The monophyly of the Myliobatiformes, as recognized by McEachran et al. [4], Carvalho et al. [3], McEachran and Aschliman [63], and Aschliman et al. [5] is confirmed and strongly supported herein (Bremer value 9) by ten synapomorphies. The phylogeny also detected a dichotomy within myliobatiforms (excluding *Hexatrygon*) as recovered by Marramà et al. [32, 36] with two main clades that partially correspond to the superfamilies Myliobatoidea and Dasyatoidea. The nature of the dichotomy is possibly linked to the different calcifications of radial cartilages, body shapes and swimming modes detected in these two groups by Schaefer and Summers [40]. For the discussion of the myliobatiform synapomorphies and the relationships within the Dasyatoidea (the benthic stingrays) we refer to the comprehensive discussion provided by Marramà et al. [32, 36].

The relationship of *Gymnura* as sister to pelagic stingrays is supported herein (with a Bremer value of 1) by six synapomorphies: short orbital region with anteriorly placed supraorbital and postorbital processes (ch. 10 [1]; CI 1.00); lateral expansion of radials in the pectoral region (ch. 28 [1]); caudal fin absent (ch. 34 [2]); first segment of the propterygium adjacent to the anterior margin of antorbital cartilage or anterior to the margin of the nasal capsule (ch. 81 [3]); ‘crustal’ calcification pattern of the radials (ch. 100[0]); and wing-like body shape, with greatly expanded pectoral fins (ch. 101 [1]; CI 1.00). This clade includes those stingrays with crustal calcification of the radials and a wing-like body shape that possibly reflect their unique oscillatory swimming mode [40]. The tree therefore shows a hypothesis that contrasts with more recent analyses in resurrecting a clade consisting of [*Gymnura* + ‘Myliobatidae’], whose relationship is only weakly supported possibly because of ambiguous character states [6]. Recent molecular analyses resolved *Gymnura* as sister to *Urolophus* [64], *Plesiobatis* [65], *Hexatrygon* [66], or placed it much closer to the base of all myliobatiforms [7]. It is noteworthy that *Gymnura* does not form the sister of pelagic stingrays but is close to the base of all myliobatiforms when characters are ordered [see Additional file 1: Figure S2]. Investigation about characters supporting its different relationships is beyond the scope of the present study.

The monophyly of pelagic stingrays (including *Promyliobatis*, *Aetomylaeus*, *Myliobatis*, *Weissobatis*, *Aetobatus*, *Rhinoptera*, and *Mobula*) is strongly-supported herein (Bremer value 11) by 16 characters, including the absence of a basihyal (ch. 19[3]); fourth and fifth ceratobranchials fused to each other (ch. 21[2]; CI 1.00); absence of median projection of the basibranchial medial plate (ch. 22[0]); cartilaginous rod on the tail (ch. 33[1]); cephalic lobes single and continuous (ch. 42[1]; CI 1.00); broad teeth on the jaws (ch. 44[1]) in pavement-like arrangement (ch. 45[1]; CI 1.00) and with a hexagonal shape (ch. 46[1]; CI 1.00); symphyseal teeth more expanded than lateral teeth (ch. 48[1]); intermediate state of the interlocking association (ch. 53[1]); smooth occlusal tooth surface (ch. 57[1]); polyaulacorhizous tooth root vascularization (ch. 60[1]; CI 1.00) with several root lobes (ch. 61[1]; CI 1.00) and showing narrow blocks in basal view (ch. 62[1]; CI 1.00); basihyal and first hypobranchial absent (ch. 76[3]; CI 1.00); and mesiodistally enlarged teeth forming a single tooth row (ch. 98[1]). *Promyliobatis* is recovered as the basalmost pelagic stingray possibly due to the presence of a single, not fragmented mesopterygium, which on the contrary is fragmented in *Myliobatis* and possibly in *Weissobatis* (ch. 27[1]) (supporting herein their sister-group relationship), and absent/fused to the scapulocoracoid in *Aetomylaeus*, *Aetobatus*, *Rhinoptera*, and *Mobula* (ch. 27[2]).

The sister group relationship between *Aetomylaeus* and the clade formed by *Myliobatis* and *Weissobatis* is supported herein by the presence of a very elongate anterior process of the Meckel’s cartilage (ch. 92[2]). The clade [*Aetobatus* + [*Rhinoptera* + *Mobula*]] is supported by four characters, including the presence of a postorbital process with a small foramen for the passage of the infraorbital lateral line canal (ch. 9[1]); lateral stays of the synarcual originating dorsal to the spinal nerve foramina (ch. 24[1]); pelvic girdle greatly arched (ch. 31[1]); and tooth roots with fine edges in basal view (ch. 62[2]; CI 1.00). The placement of *Aetobatus* as sister to [*Rhinoptera* + *Mobula*] is problematic since it contrasts with molecular data that suggest a closer relationship of *Aetobatus* with *Myliobatis* and *Aetomylaeus* [7, 64]. Finally, the sister relationship between *Rhinoptera* and *Mobula* is supported by the presence of an anterior processes of the neurocranium (ch. 5[1]; CI 1.00); absence of a preorbital processes (ch. 6[1]; CI 1.00); absence of lateral expansions in pectoral radials (ch. 28[0]); a very elongated median prepelvic process (ch. 30[1]); and tooth association loosely interlocking (ch. 53[0]). Although the resolution of the tree is reduced after a bootstrap analysis with 1000 replicates [Additional file 1: Figure S2], the relationships among pelagic stingrays are basically the same with the basalmost position of *Promyliobatis* still retrieved. Despite the high level of homoplasy, the recovering of similar phylogenetic hypotheses in pelagic stingrays using different approaches may suggest that the data and sampling of taxa are adequate, and that homoplastic characters can be considered diagnostic for them [67].

The inclusion of fossil taxa based on isolated teeth or dental plates only produced some phylogenetic hypotheses in which the relationships among pelagic stingrays are poorly defined [Additional file 1: Figure S3]. The position of *Promyliobatis* within the Myliobatiformes becomes unstable, and only when characters are considered ordered the analysis results in a tree, which is similar to that presented by Claeson et al. [3]. In this latter tree *Myliobatis* is paraphyletic and *Promyliobatis* is highly nested and apparently more derived than most of the extant and fossil eagle rays. It must be pointed out that coding for taxa based on isolated teeth only covers 22 out of 103 characters (about 80% of data missing) possibly resulting in lower resolution and, generally, in different hypotheses with respect to those of Claeson et al. [3], Adnet et al. [9] and Blanco [42]. The inclusion of taxa with a lot of missing characters coupled with the extremely high morphological variation in tooth morphology in extant and extinct *Myliobatis* taxa [21] may be therefore not useful to conclusively resolve the relationships among pelagic stingrays.

## Discussion

*Promyliobatis gazolai* has a number of features that clearly support its inclusion within the order Myliobatiformes, including the absence of a rostral cartilage, presence of a thoracolumbar synarcual and a serrated tail sting [3, 5, 68]. *Promyliobatis* can be considered as a genuine member of the pelagic stingrays based on the absence of a basihyal, fourth and fifth ceratobranchials that are fused to each other, the absence of a median projection of the basibranchial medial plate, the presence of cephalic lobes, a crushing pavement-like dentition with symphyseal teeth that are more expanded than the lateral teeth, and a polyaulacorhizous root vascularization pattern.

### Skeletal morphological comparison

The morphological and phylogenetic analysis of the stingrays that includes only the holomorphic fossil taxa, detected *Promyliobatis gazolai* as sister to all the other pelagic stingrays because of the presence of some plesiomorphic features, including a single, not fragmented mesopterygium (fragmented in *Myliobatis* and possibly *Weissobatis*, absent/fused to scapulocoracoid in *Aetomylaeus*, *Aetobatus*, *Rhinoptera*, and *Mobula*; [3, 16]). The absence of a considerably arched pelvic girdle excludes any alignment with *Aetobatus*, *Rhinoptera*, or *Mobula*, whereas the absence of the anterior process of the neurocranium and the median prepelvic process, as well as the presence of lateral expansions in pectoral radials are useful to separate *P*. *gazolai* from the cownose (*Rhinoptera*) and devil rays (*Mobula*). Additionally, the morphology of the anterior margin of the cephalic lobes is useful to differentiate *Promyliobatis* (single and continuous) from *Aetobatus* (single with indentation), *Rhinoptera*, and *Mobula* (completely separated into two distinct cephalic fins). The overall body plan of *Promyliobatis* is therefore more consistent with that of the eagle rays (including *Aetomylaeus*, *Myliobatis*, *Weissobatis*) although several differences can be recognized. Other than for the general shape of the disc (anterior and posterior margins nearly straight in *Promyliobatis*, concave or convex in other genera), the Bolca taxon can be distinguished from the other eagle rays by having different meristic counts (see Table 2), an arched metapterygium (straight in *Weissobatis*) and the absence of the compagibus laminam (the latter is present in *Aetomylaeus*, *Aetobatus*, and some *Myliobatis* species).

**Table 2:** Selected skeletal and dental characters in selected pelagic durophagous stingray genera

		*Promyliobatis*	*Weissobatis*	*Myliobatis*	*Aetomylaeus*	*Aetobatus*
Skeletal characters						
Rostral radials		12–15	?	7–17	7–10	10–16
Propterygial radials (excl. rostral)		35	25	19–23	13–16	11–15
Mesopterygial radials		10–12	?	16–23	24–28	30–37
Metapterygial radials		40	?	41–52	41–53	55–66
Total pectoral radials (excl. rostral)		87	?	79–92	79–92	89–116
Pelvic radials		22–23	?	17–25	14–19	14–19
Monospondylous trunk vertebrae (excl. synarcual)		20–22	?	24–32	31–42	31–41
Diplospondylous vertebrae (anterior to sting)		148	?	34–48	5–20	13–31
Diplospondylous vertebrae (posterior to sting)		50	?	36–47	34–36	25–33
Total vertebrae		218	85 +?	108–117	80–86	80–97
Number of stings		1	1	1–3	0–2	1–2
Compagibus laminam		absent	present?	absent/present	present	present
Continuity pectoral - rostral radials		continuous	?	continuous	interrupted	interrupted
Rostral radials less developed than pectoral radials		yes	?	no	yes	yes
Mesopterygium		single	fragmented?	fragmented	absent/fused	absent/fused
Puboischiadic bar		slightly arched	?	slightly arched	slightly arched	greatly arched
Tooth characters						
Medial teeth width/length ratio	Upper	3.6–4.1	6.0	3.0–7.0	4.0–18.0	10.0–13.0
	Lower	3.8–4.5	6.0	3.0–5.0	4.0–18.0	6.0–11.0
Medial teeth shape	Upper	slightly bent	moderately bent	straight to moderately bent	straight to bent to M-shaped (adult)	straight to M-shaped
	Lower	straight to slightly bent	almost straight	straight to moderately bent	straight to strongly bent	V-shaped
Lateral tooth row number	Upper	2	1	2–3	0–2	0
	Lower	2	1	2–4	0–2	0
Posterior tooth row number		1	1	1	0–1	0
Lateral teeth shape		hexagonal/rhomboidal	hexagonal/square	hexagonal/square/lozenge	square/lozenge	–

Data from Nishida [1], Hovestadt and Hovestadt-Euler [21], White et al. [61, 62, 82, 83], White [16], Hall et al. [59]. Although the mesopterygium is absent (fused to scapulocoracoid) in *Aetomylaeus* and *Aetobatus*, Hall et al. [59] considered radials not distinctly articulated with the propterygium or metapterygium as ‘mesopterygial radials’

### Tooth morphology

The filter-feeding devil rays of the genus *Mobula* are easily separated from *Promyliobatis* by the absence of large crushing dentitions, which are replaced by small numerous peg-like teeth that reflect their loss of mastication [9]. Several tooth characters are also useful to distinguish *Promyliobatis* from *Aetobatus* and *Rhinoptera* [Additional file 1: Figure S4]. *Aetobatus* can be distinguished from *Promyliobatis* in the absence of lateral teeth and presence of a single row of symphyseal teeth, which are M-shaped in upper (in adult), and strongly V-shaped in lower jaws [20, 21, 39]. The cownose stingray *Rhinoptera* possesses some of the hexagonal lateral teeth, which are mesio-distally very enlarged, although less than the symphyseal teeth, in a condition which is also found in the extinct taxa *Brachyrhizodus* and *Igdabatis* [10, 20, 39].

As pointed out by Carvalho et al. [3], the dentition of *Promyliobatis* is more similar to that of *Myliobatis* or *Aetomylaeus*, with much wider central symphyseal teeth articulating laterally in pavement-like arrangement with smaller lateral and posterior teeth. Hovestadt and Hovestadt-Euler [21] recognized up to 16 kinds of tooth variations within *Aetomylaeus* (four within the ‘*Pteromylaeu*s’ morphotype, and 12 within the ‘*Aetomylaeus*’ morphotype). Although high intra- and interspecific variation is present in *Aetomylaeus*, this genus usually exhibits distally extended, hexagonal symphyseal teeth having labial and lingual margins that are slightly to strongly lingually curved, two rows of obliquely square to lozenge-shaped labio-lingually extended lateral teeth, and a similar posterior row in both upper and lower jaws [Additional file 1: Figure S4]. However, if we exclude the shape of the lateral teeth (squared to lozenge in *Aetomylaeus*, hexagonal to rhomboidal in *Promyliobatis*), the number, shape and proportions of symphyseal teeth appear not useful to clearly separate *Promyliobatis* from *Aetomylaeus* (see Table 2), also considering that juveniles of *Aetomylaeus* possess a *Myliobatis*-like tooth morphologies [21], which is also typical of *Promyliobatis*. In our opinion, tooth characters are instead completely useless for clearly distinguishing *Promyliobatis* from *Myliobatis* (with nine different morphological variations within the extant genus being recognized), leading Hovestadt and Hovestadt-Euler [21] to include *P. gazolai* within what they defined to as *Myliobatis* Variation 1 based on its tooth morphology. Finally, *Weissobatis* can be distinguish from *Promyliobatis* by having a different width/length ratio of symphyseal teeth (about 6.0 vs 3.6–4.5), more pronounced curvature of the upper teeth, and presence of only one lateral tooth row (two in *Promyliobatis*). According to Hovestadt and Hovestadt-Euler [21] the ornamentation of the interlocking mechanism in isolated teeth might be useful to distinguish teeth of *Aetomylaeus* (fine to coarse scattered costules), *Myliobatis* (coarse vertically directed costules) and *Aetobatus* (horizontally directed furrows). However, it was not possible to examine this character in *Promyliobatis* and the condition is unknown in *Weissobatis*.

On the other hand, *Promyliobatis* can easily be distinguished from other Eocene ‘eagle ray’ genera in the absence of thick and strongly granulose enameloid that characterizes the occlusal face of teeth of *Leidybatis* Cappetta, 1986 [69], or of the pitted enameloid of *Garabatis* Cappetta, 1993 [70], or the transversely deep hollows on the occlusal surface of teeth of *Aktaua* Case et al., 1996 [71]. The strongly arched symphyseal teeth of the Eocene *Pseudoaetobatus* Cappetta, 1986 [69] mostly resemble those of *Aetobatus*, but this extinct genus also possesses asymmetrical lateral teeth with tapered and posteriorly curved distal margins [20]. Finally, the dentitions of the Paleogene genera *Archaeomanta* Herman, 1979 [72], *Burnhamia* Cappetta, 1976 [73], *Eomobula* Herman, Hovestadt-Euler and Hovestadt, 1989 [74], *Eoplinthicus* Cappetta and Stringer 2002 [75], *Plinthicus* Cope, 1869 [76] and *Sulcidens* Underwood, Kolman and Ward, 2007 [77] clearly show characters typical of Rhinopteridae or Mobulidae [9, 10, 20].

More than 60 Eocene eagle ray species are known based on isolated teeth or tooth plates, of which about 45 have been referred to the wastebasket genus *Myliobatis* [21]. However, it seems that there are no unambiguous characters, which clearly allow defining the different species within the genus, leading Hovestadt and Hovestadt-Euler [21] to include them within the *Myliobatis* or *Aetomylaeus* variations. In this perspective, we do not exclude that most of them, including indeterminate ‘*Myliobatis’* material from shallow Tethyan regions [78], could belong to *Promyliobatis*. However, this is not possible to conclusively determine until articulated skeletal material associated with tooth plates of these species will be found.

### Evolutionary remarks

It is assumed that myliobatiforms diverged from their sister group, the panrays (today represented by *Zanobatus*) around 150 million years ago [64, 66] since “*Dasyatis*” *speetonensis*, the oldest stingray possibly closely related to *Hexatrygon*, is Hauterivian (Early Cretaceous) in age [79]. Subsequently, myliobatioforms experimented a diversification during the late Late Cretaceous [64, 66]. Divergence time estimates place the origin and radiation of the pelagic durophagous stingrays around or slightly before the K-Pg boundary, coincident with the immediate niches filling scenario of the benthic K-Pg survivors and their exploitation by durophagous stingrays [64, 66, 80]. After the appearance and initial radiation of planktivorous taxa during late Paleocene-early Eocene [77], a second wave of radiation occurred at the Oligocene-Miocene boundary within pelagic stingrays when the filter-feeding devil rays Mobulidae possibly separated from the Rhinopteridae [64, 66].

The fossil record of pelagic stingrays is extensive and widespread, including more than 150 fossil species dating back at least to the Late Cretaceous (Campanian) of Texas and Spain [10, 20]. This might suggest a Late Cretaceous northern Hemisphere (Tethyan and North Atlantic) origin for durophagous stingrays, as also hypothesized for other batomorph lineages (e.g. skates [81, 82]). Pelagic stingrays are considerably well diversified in the Paleocene and early Eocene and numerous species have been recovered from warm water Neogene deposits of the Mesogean Sea up to now [20]. However, it seems there is still disagreement between the fossil record, divergence estimates and the different phylogenetic hypotheses attempted to understand the relationships and evolutionary trends of pelagic stingrays. For example, the monophyly of *Myliobatis* species is supported by molecular analysis [65, 80], but not by morphological characters [5, 10]. *Igdabatis* is placed sister to a clade consisting of [Rhinopteridae + Mobulidae] in Claeson et al. [10], sister to *Rhinoptera* in Blanco [42] and to *Myliobatis vuornensis* in the present study [see Additional file 1: Figure S3]. *Burnhamia davisei* was recovered deeply nested within or sister to *Rhinoptera* [10], or sister to Mobulidae [9, 42]. *Brachyrhizodus* was identified as the sister to living Mobulidae [10], or the sister to a clade formed by [*Rhinoptera* + *Igdabatis*] [42], or, alternatively, in a more basal position within the pelagic stingrays ([9] and this study). The recovering of *Sulcidens* in polytomous relationships even in our analyses [see Additional file 1: Figure S3] makes still uncertain its relative phylogenetic position [77]. It is therefore likely that this general disagreement may reflect the retention or re-derivation of ancestral tooth character states within some lineages as already suggested by Aschliman [6].

## Conclusions

The skeletal and dental morphology of *Promyliobatis gazolai* supports the previous hypothesis of Jaekel [35], who identified it to represent a distinct Eocene taxon. The phylogenetic analysis presented here (including only holomorphic specimens) recovers *Promyliobatis* as the basalmost pelagic stingray due to the absence of some derived features (e.g. a mesopterygium fragmented or fused to the scapulocoracoid) that characterize the other eagle, cownose and devil rays. Considering the basal position of *Promyliobatis*, it is likely that the appearance of this genus can be linked to the radiation and exploitation of benthic resources by pelagic durophagous stingrays after the end-Cretaceous event. Moreover, the absence of unambiguous tooth characters that could distinguish *Promyliobatis* undoubtedly from the living eagle rays *Aetomylaeus* and *Myliobatis* highlights the importance of parsimony in the identification and erection of new species of pelagic durophagous stingrays based on isolated teeth or dental plates only.

## Additional file

Additional file 1: Mosaic of plesiomorphic and derived characters in an Eocene myliobatiform batomorph (Chondrichthyes, Elasmobranchii) from Italy defines a new, basal body plan in pelagic stingrays. **Figure S1. a** Location and geological map of the Bolca area. **b** Stratigraphic section of the Pesciara site. Adapted and modified from Papazzoni and Trevisani (2006) and Trevisani (2015). **Figure S2.** Additional phylogenetic analyses showing the relationships of †*Promyliobatis gazolai* (de Zigno, 1882) within the Myliobatiformes using living and fossil taxa based on holomorphic specimens. Numbers indicate the bootstrap values. **Figure S3.** Phylogenetic hypotheses showing the relationships of †*Promyliobatis gazolai* (de Zigno, 1882) within the Myliobatiformes also including fossil taxa based on isolated teeth or dental plates previously used by Claeson et al. (2010). The trees are from analyses including all taxa, with benthic stingrays (Dasyatoidea) condensed as a single outgroup taxon. ‘*A*.’ stands for the *Aetobatus* species, ‘*M*.’ indicates *Myliobatis* species. **Figure S4.** Upper and lower dental plates of extant pelagic durophagous stingray genera used for comparisons. **a** *Myliobatis aquila*; photo: courtesy of Dr. D. Hovestadt. **b** *Aetomylaeus* sp., EMRG-Chond-T-58. **c** *Aetobatus* sp., EMRG-Chond-T-60. **d** *Rhinoptera* sp., EMRG-Chond-T-59. Scale bars = 10 mm. (DOCX 2799 kb)
