# Supplementary material for: Mosaic of plesiomorphic and derived characters in an Eocene myliobatiform batomorph (Chondrichthyes, Elasmobranchii) from Italy defines a new, basal body plan in pelagic stingrays
Source: Zoological Lett. 2019 Apr 25;5:13. doi: 10.1186/s40851-019-0128-0 (PMC6485097; doi:10.1186/s40851-019-0128-0)
Supplement: Supplementary file 1 — Mosaic of plesiomorphic and derived characters in an Eocene myliobatiform batomorph (Chondrichthyes, Elasmobranchii) from Italy defines a new, basal body plan in pelagic stingrays. Figure S1. a Location and geological map of the Bolca area. b Stratigraphic section of the Pesciara site. Adapted and modified from Papazzoni and Trevisani (2006) and Trevisani (2015). Figure S2. Additional phylogenetic analyses showing the relationships of †Promyliobatis gazolai (de Zigno, 1882) within the Myliobatiformes using living and fossil taxa based on holomorphic specimens. Numbers indicate the bootstrap values. Figure S3. Phylogenetic hypotheses showing the relationships of †Promyliobatis gazolai (de Zigno, 1882) within the Myliobatiformes also including fossil taxa based on isolated teeth or dental plates previously used by Claeson et al. (2010). The trees are from analyses including all taxa, with benthic stingrays (Dasyatoidea) condensed as a single outgroup taxon. ‘A.’ stands for the Aetobatus species, ‘M.’ indicates Myliobatis species. Figure S4. Upper and lower dental plates of extant pelagic durophagous stingray genera used for comparisons. a Myliobatis aquila; photo: courtesy of Dr. D. Hovestadt. b Aetomylaeus sp., EMRG-Chond-T-58. c Aetobatus sp., EMRG-Chond-T-60. d Rhinoptera sp., EMRG-Chond-T-59. Scale bars = 10 mm. (DOCX 2799 kb) [file 40851_2019_128_MOESM1_ESM.docx]

**Additional file 1**

Mosaic of plesiomorphic and derived characters in an Eocene myliobatiform batomorph (Chondrichthyes, Elasmobranchii) from Italy defines a new, basal body plan in pelagic stingrays

Giuseppe Marramà^1^*, Giorgio Carnevale^2^, Gavin J. P. Naylor^3^ and Jürgen Kriwet^1^

^1^University of Vienna, Department of Palaeontology, Althanstrasse 14, 1090, Vienna, Austria; e-mails: giuseppe.marrama@univie.ac.at; juergen.kriwet@univie.ac.at

^2^ Università degli Studi di Torino, Dipartimento di Scienze della Terra, Via Valperga Caluso 35, 10125 Torino, Italy; e-mail: giorgio.carnevale@unito.it

^3^ University of Florida, Florida Museum of Natural History, 1659 Museum Road, 32611 Gainesville, USA; e-mail: gjpnaylor@gmail.com

*Corresponding author

**Geological setting**

Lithological and sedimentological features of the slabs suggest that the two specimens forming the object of this study were collected from the fossiliferous layers of the Pesciara site of the Bolca Konservat-Lagerstätte, located in the Lessini Mountains (southern Alps), about 2 km north-east of the village of Bolca, Verona Province, north-eastern Italy (Fig. S1). The stratigraphic sequence of the Pesciara site is traditionally referred to as the ‘Calcari Nummulitici’, an informal unit of Eocene age widely distributed in north-eastern Italy (Papazzoni & Trevisani 2006). The entire succession is formed by a nearly 20-m-thick alternation of finely laminated micritic limestones (containing fishes, plants and invertebrates) and coarse-grained biocalcarenite-biocalcirudite (with a rich benthic fauna). Studies based on the benthic foraminiferan content suggest that the fish-bearing limestones of the Pesciara belong to the †*Alveolina dainelli* Zone or SBZ 11 Biozone (Papazzoni & Trevisani 2006; Papazzoni et al. 2014), corresponding to the late Cuisian (late Ypresian, about 49 Ma). A quantitative palaeoecological analysis by Marramà et al. (2016) revealed that the Pesciara fish assemblage was characterized by a sharp oligarchic structure dominated by zooplanktivorous fishes, especially sardines and allies. The related taphonomic analysis confirmed that the sediments were deposited in an intraplatform basin in which bottom anoxia and the development of a biofilm promoted the high-quality preservation of the fossils (Papazzoni & Trevisani 2006; Marramà et al. 2016).


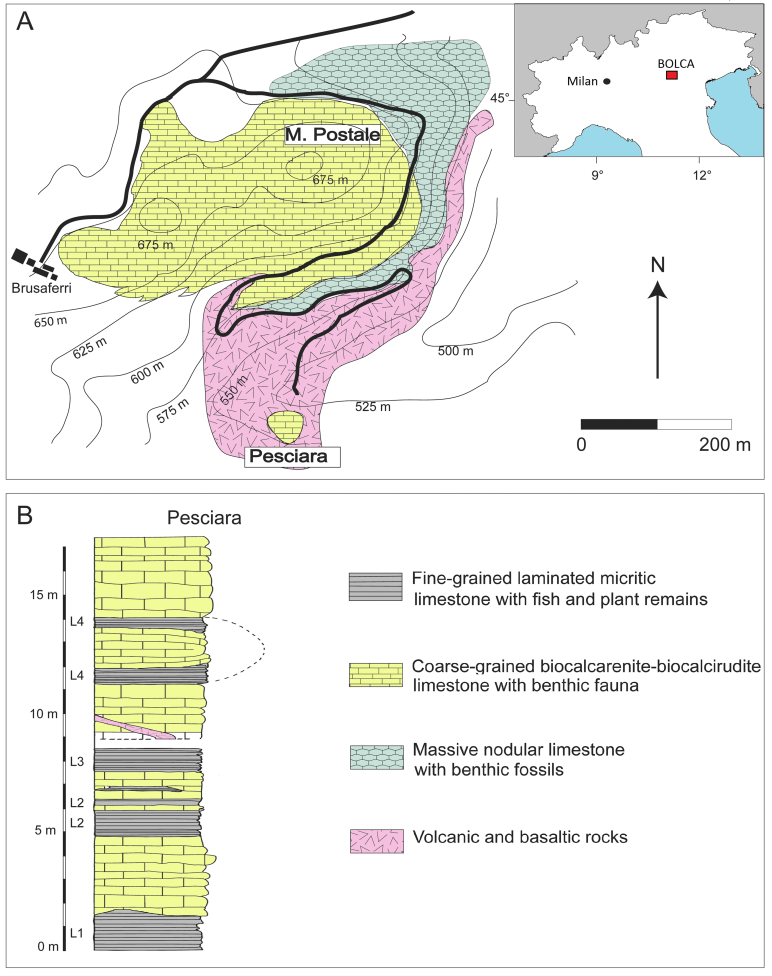


**Fig. S1** **a** Location and geological map of the Bolca area. **b** Stratigraphic section of the Pesciara site. Adapted and modified from Papazzoni and Trevisani (2006) and Trevisani (2015).

**
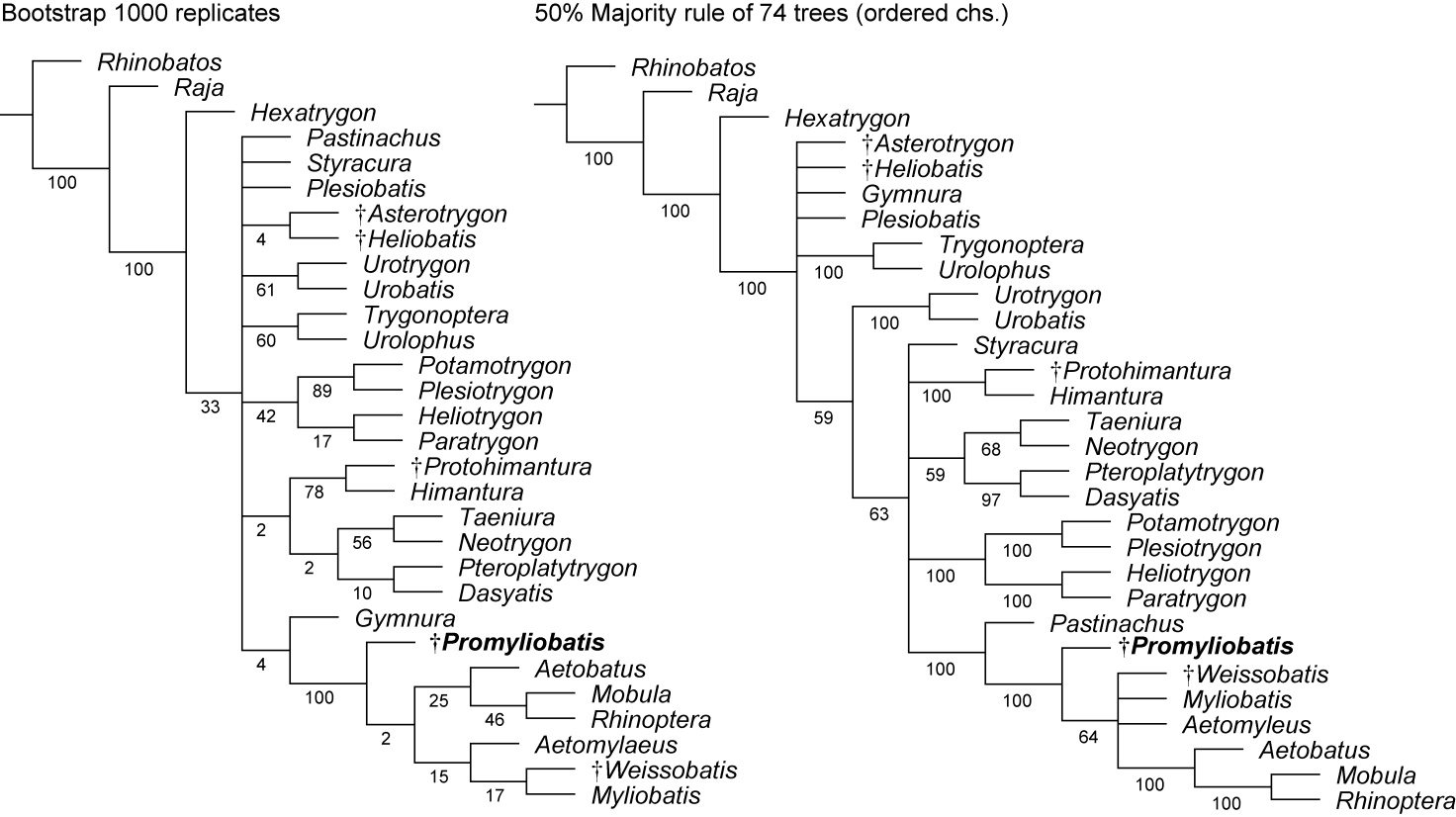
**

**Fig. S2** Additional phylogenetic analyses showing the relationships of †*Promyliobatis gazolai* (de Zigno, 1882) within the Myliobatiformes using living and fossil taxa based on holomorphic specimens. Numbers indicate the bootstrap values.

**
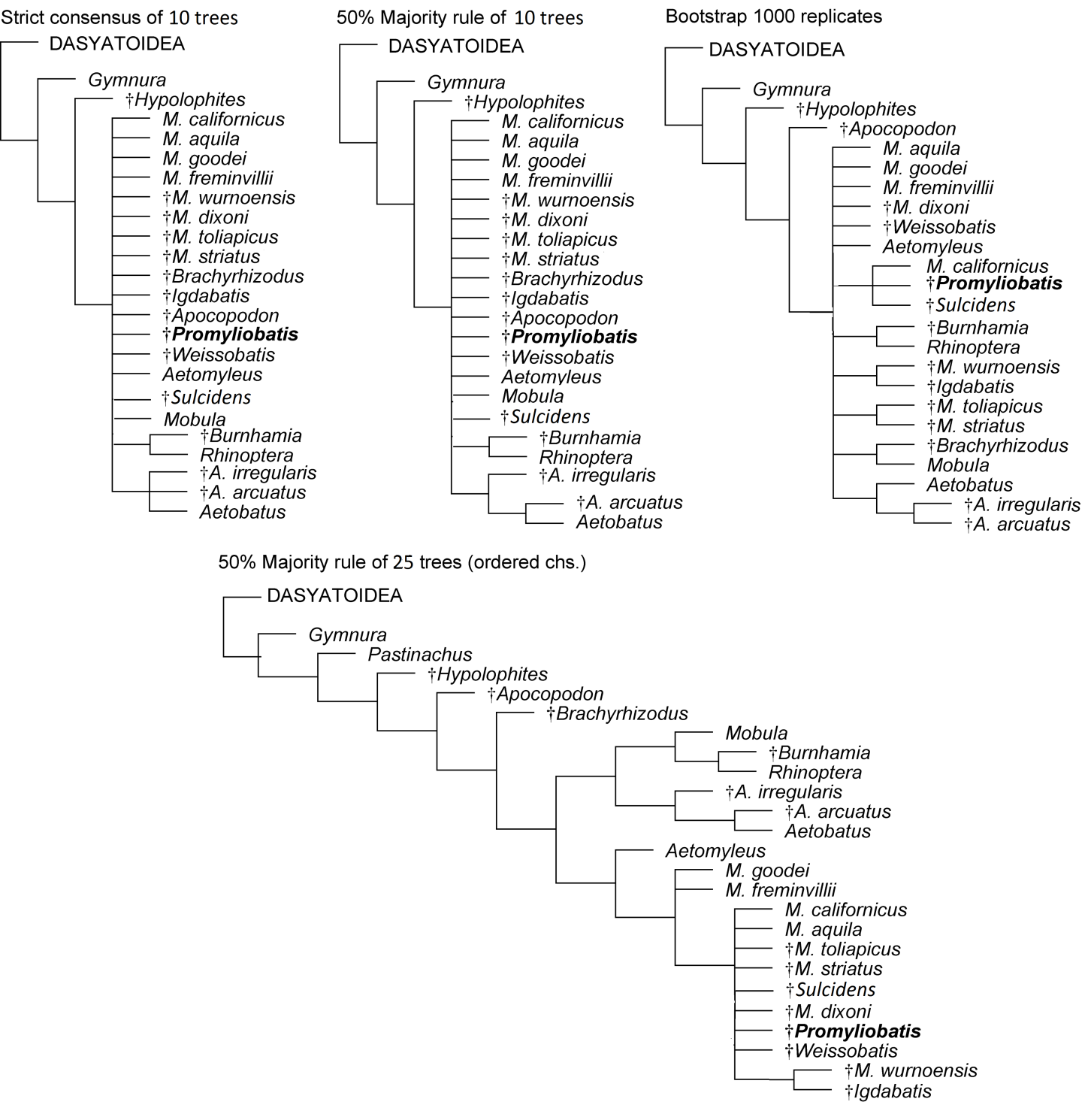
**

**Fig. S3** Phylogenetic hypotheses showing the relationships of †*Promyliobatis gazolai* (de Zigno, 1882) within the Myliobatiformes also including fossil taxa based on isolated teeth or dental plates previously used by Claeson et al. (2010). The trees are from analyses including all taxa, with benthic stingrays (Dasyatoidea) condensed as a single outgroup taxon. ‘*A*.’ stands for the *Aetobatus* species, ‘*M*.’ indicates *Myliobatis* species.

**
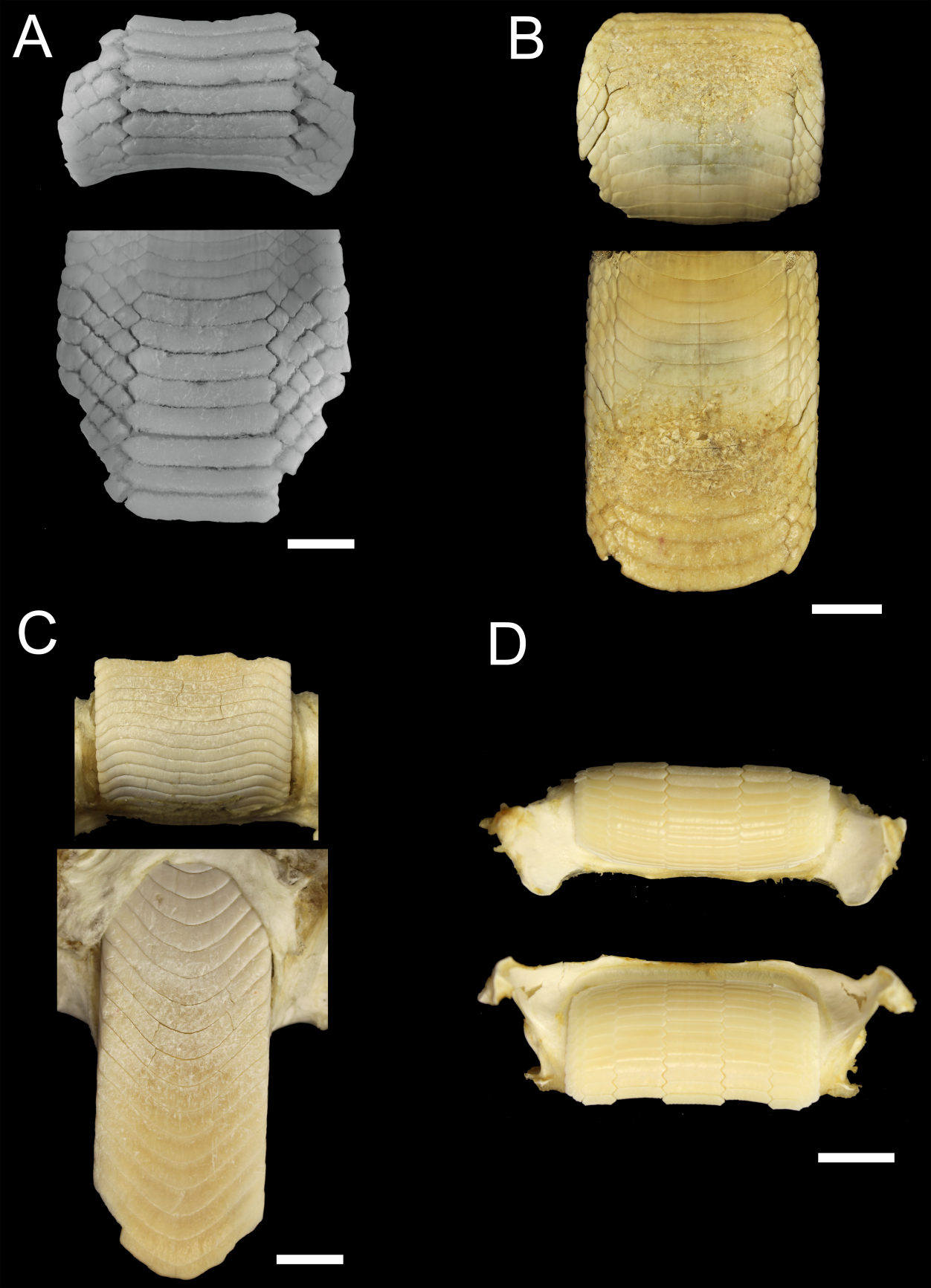
**

**Figure S4.** Upper and lower dental plates of extant pelagic durophagous stingray genera used for comparisons. **a** *Myliobatis aquila*; photo: courtesy of Dr. D. Hovestadt. **b** *Aetomylaeus* sp., EMRG-Chond-T-58. **c** *Aetobatus* sp., EMRG-Chond-T-60. **d** *Rhinoptera* sp., EMRG-Chond-T-59. Scale bars = 10 mm.

**Appendix A.** List of morphological characters used for the phylogenetic analysis, based on Marramà et al. (2018a*,* 2018b).

1. Tubules of subpleural components of hyomandibular lateral line canals: (0) not branched at extremities; (1) extremities dichotomously branched

2. Subpleural components of the hyomandibular lateral line canals: (0) posterior branch extends caudally more or less parallel to longitudinal body axis; (1) posterior branch inflects towards midline to form a lateral hook; (2) posterior branch inflects to continue anteriorly almost parallel to anterior branch, forming a large indentation

3. Suborbital components of infraorbital lateral line canals: (0) projecting posteriorly lateral to mouth; (1) projecting posteriorly lateral to mouth and anteriorly lateral to nasal openings; (2) forming a complex web−like pattern on lateral aspects of the anteroventral disc region

4. (04 of CMG) Scapular loops formed by scapular components of trunk lateral line canals: (0) absence of loops; (1) presence of scapular loops

5. Anterior process of neurocranium: (0) absent; (1) present

6. Preorbital process: (0) present; (1) absent

7. Preorbital canal for passage of superficial ophthalmic nerve: (0) dorsally located; (1) anteriorly located

8. Foramen for the optic (II) nerve: (0) moderately sized; (1) very enlarged

9. Postorbital process of neurocranium: (0) infraorbital lateral line canal separates postorbital process from small, anterior triangular outgrowth (supraorbital process) of the supraorbital crest; (1) postorbital process with small foramen for passage of infraorbital lateral line canal. **Remarks**: this character has been changed to polymorphic (0/1) for *Aetomylaeus* following Aschliman (2014).

10. Extent of orbital region: (0) orbital region of neurocranium long; (1) shortened orbital region with more anteriorly placed supraorbital and postorbital process

11. Postorbital process: (0) without ventrolateral projection; (1) continuing ventrolaterally to form a cylindrical projection

12. Ventrolateral expansion of nasal capsules: (0) nasal capsules laterally expanded; (1) nasal capsules ventrolaterally expanded

13. Articulation between hyomandibula and Meckel’s cartilage: (0) hyomandibulae directly attached to lower jaws; (1) hyomandibulae articulating with lower jaws through strong, stout ligament (hyomandibular−Meckelian ligament) at distal tip

14. Angular cartilages: (0) absence of angular cartilages within hyomandibular−Meckelian ligament; (1) presence of angular cartilages within ligament

15. Secondary hyomandibular cartilages: (0) absent; (1) present

16. Symphysial fusion of upper and lower jaws: (0) antimeres separate at symphysis; (1) both antimeres of jaws symphysially fused. **Remarks**: Following Aschliman (2014) the character has been recoded as polymorphic for *Myliobatis*, since *M. freminvillei* exhibits the plesiomorphic condition (antimeres unfused).

17. Mandibular width at symphysis: (0) lower jaws slender at symphysis; (1) lower jaws symphysially thickened

18. Lateral projections of lower jaws: (0) absent; (1) present

19. Basihyal cartilage: (0) basihyal laterally elongated, fused to first hypobranchialis; (1) basihyal a single element, but separate from first hypobranchials; (2) basihyal separate from first hypobranchials but fragmented into more than one component; (3) basihyal absent

20. Fusion of ventral pseudohyoid and first ceratobranchial: (0) absent; (1) present

21. Arrangement of posterior ceratobranchials: (0) separate from each other; (1) ankylosis between fourth and fifth ceratobranchials; (2) fourth and fifth ceratobranchials fused to each other.

22. Median projection of the basibranchial medial plate: (0) absent; (1) present

23. Articulation between fifth epi− and ceratobranchial elements to scapulocoracoid: (0) close together; (1) widely separated

24. Lateral stay of synarcual: (0) originates ventral to spinal nerve foramina; (1) originates dorsal to spinal nerve foramina; (2) contacting synarcual both dorsally and ventrally to foramina

25. Fossa on dorsal scapular region: (0) absent; (1) present

26. Contact between pro- and mesopterygium in the pectoral fin: (0) present; (1) absent.

27. Distinct components of the mesopterygium: (0) mesopterygium single element; (1) fragmented; (2) missing altogether. **Remarks**: since the mesopterygium is absent (possibly fused to scapulocoracoid) in *Aetomylaeus*, the state has been changed from (1) to (2) following White (2014), whereas it has been coded (1, fragmented) in *Weissobatis* following the drawing in Hovestadt & Hovestadt-Euler (1999, fig. 3).

28. Lateral expansion of radials in pectoral region: (0) absent; (1) present

29. External margin of mesopterygium: (0) more or less straight, not fused to radials; (1) undulated, not fused to radials; (2) highly sinuous, appearing to be fused with articulating radial elements

30. Median prepelvic process: (0) absent or weakly developed; (1) very elongated. **Remarks**: the presence of a greatly elongated median prepelvic process of the puboischiadic bar has been reported also for *Rhinoptera* and *Mobula* (see Aschliman 2014) and their state recoded consequently.

31. Pelvic girdle shape: (0) not arched or only moderately so; (1) greatly arched

32. Dorsal fin: (0) present; (1) absent

33. Cartilaginous rod in tail: (0) absent; (1) present

34. Caudal fin: (0) present; (1) reduced to tail−folds; (2) absent

35. Adductor mandibulae complex: (0) without posteromedial extension; (1) posteromedial extension present

36. Spiracularis muscle: (0) projecting ventrally to insert on either palatoquadrate, Meckel’s cartilage, and or hyomandibula; (1) projecting ventrally and posteriorly beyond hyomandibulae and both sets of jaws to insert dorsal to coracomandibularis; (2) projecting ventrally and posteriorly beyond hyomandibulae and both sets of jaws to insert ventral to coracomandibularis

37. Depressor mandibularis muscle: (0) present; (1) absent

38. Coracohyoideus muscle: (0) not connected at midline; (1) connected at midline

39. Urea retention: (0) urea retained in blood; (1) urea excreted in urine

40. Rectal gland: (0) present; (1) reduced

41. Spiracular tentacle: (0) absent; (1) present

42. Cephalic lobes: (0) absent; (1) single and continuous; (2) single with an indentation; (3) paired

43. Nasal curtain: (0) not reaching mouth region; (1) extending posteriorly as far as mouth opening

44. Tooth type in both upper and lower jaws: (0) minute; (1) broad

45. Arrangement of teeth in both upper and lower jaws: (0) arranged in separate diagonal rows or ribbons; (1) horizontal conveyor or pavement−like arrangement

46. Tooth shape: (0) square to rounded; (1) hexagonal, six distinct sides; (2) rectangular with posteriorly deflected lateral margins.

47. Lateral teeth: (0) present; (1) absent

48. Differentiation of median teeth from lateral teeth: (0) median and lateral teeth are similar; (1) median teeth relatively expanded

49. Differentiation among lateral teeth: (0) lateral teeth unexpanded; (1) some lateral teeth expanded

50. Relative amount of curvature in expanded lower teeth: (0) straight and uncurved; (1) moderately curved; (2) strongly curved. **Remarks**: because of the extremely high ontogenetic, inter- and intraspecific variation in tooth morphologies in *Myliobatis* and *Aetomylaeus* (see Hovestadt & Hovestadt-Euler 2013) the coding for chs. 50 to 53 cannot be restricted to one single state for these taxa, so we prefer to consider polymorphic ([0&1] in chs 50, 51, 53; and [1&2] in ch. 52) their condition. Moreover, following the description of Claeson et al*.* (2010) the state of ch. 50 for *Rhinoptera* has been changed from (1) to (0).

51. Upper tooth curvature: (0) uncurved; (1) curved

52. Direction of tooth curvature: (0) concave; (1) flat/horizontal; (2) convex

53. Tooth association: (0) loosely interlocking; (1) sometimes loosely interlocking or tightly interlocking; (2) tightly interlocking

54. Tooth Interlocking mechanism: (0) overlapping; (1) tongue and groove; (2) no direct contact

55. Shape of interlocking tongue: (0) bulbous; (1) short shelf; (2) long shelf. **Remarks**: this character has been recoded has unknown for *Protohimantura*, due to an erroneous coding in the previous analysis (Marramà et al. 2018a, 2018b).

56. Crown height: (0) high, the crown height exceeds root depth on unworn teeth; (1) low crown

57. Occlusal surface: (0) cusped; (1) smooth; (2) depressed

58. Crown shape in anterior or posterior view: (0) straight; (1) domed; (2) deep

59. Lateral margins: (0) not pinched; (1) pinched

60. Root type: (0) holaulacorhizous; (1) polyaulacorhizous

61. Number of roots: (0) 2 roots; (1) more than 2 roots. **Remarks**: we only included two states instead of the original three of Claeson et al. (2010) since their state (1) (3 to 4 roots) is only characteristic of *Brachyrhizodus*, which is not included here. States are therefore re-coded consequently. In the second analysis with all extinct taxa we used the original three states of Claeson et al*.* (2010).

62. Roots in basal view: (0) triangles; (1) narrow blocks; (2) fine edges. **Remarks:** we only included three states instead of the original four of Claeson et al*.* (2010) since the state (1) of Claeson et al. (2010) (wide blocks) is only characteristic of extinct myliobatids, which are not included here. States are therefore re-coded consequently. In the second analysis with all extinct taxa we used the original four states of Claeson et al. (2010).

63. Distance between roots: (0) narrower than root laminae; (1) broad, groove wider than root laminae. **Remarks:** the polarity of the states follows here Claeson et al. (2010) at pag. 666, contrary to Claeson et al. (2010) at pag. 674, and consequently Marramà et al. (2018a, 2018b), in which the polarity was accidentally inverted. The character is polymorphic in *Myliobatis* (see Claeson et al. 2010).

64. Inclination of roots: (0) no inclination; (1) offset and step−like; (2) long and strongly inclined

65. Root groove position: (0) regularly spaced between laminae; (1) irregularly spaced between laminae

66. Levator and depressor rostri muscles: (0) absent; (1) present

67. Serrated tail stings: (0) absent; (1) present.

68. Placoid scales: (0) uniformly present; (1) limited; (2) absent.

69. Thorns: (0) present; (1) absent.

70. Pulp cavities in tooth roots: (0) large; (1) broad and elongated; (2) small; (3) absent

71. (Tooth vascularization: (0) orthodont; (1) osteodont; (2) modified osteodont.

72. (21 of ASC) Infraorbital loop of suborbital and infraorbital canals: (0) absent; (1) present and forming a simple posterolaterally directed loop; (2) present and forming a complex reticular pattern or a number of loops; (3) the loop is directed to the anterior

73. (26 of ASC, modified) Rostral cartilage: (0) complete; (1) vestigial or absent.

74. Postorbital process: (0) narrow; (1) very broad and shelf-like.

75. Jugal arch: (0) present; (1) absent.

76. Basihyal and first hypobranchial: (0) both present and unsegmented; (1) basihyal is segmented; (2) basihyal is absent; (3) basihyal and first hypobranchial cartilages absent

77. Suprascapulae: (0) articulates with vertebral column; (1) fused medially to synarcual (= pectoral arch); (2) fused medially and laterally to synarcual.

78. Ball and socket articulation between scapular process and synarcual: (0) absent; (1) present.

79. Second (thoracolumbar) synarcual: (0) absent; (1) present.

80. Ribs: (0) present; (1) absent.

81. Segmentation of propterygium: (0) posterior to mouth, (1) proximal segment of propterygium of pectoral girdle is between mouth and antorbital cartilage; (2) the first segment is adjacent to the nasal capsule; (3) the first segment is adjacent to anterior margin of antorbital cartilage or anterior to margin of nasal capsule

82. Pseudosiphon: (0) present; (1) absent

83. Dorsal marginal clasper cartilage: (0) lacks medial flange; (1) possesses medial flange

84. Dorsal terminal cartilage: (0) smooth margin; (1) crenate margin

85. Cartilage forming component claw: (0) present; (1) absent; (2) cartilage embedded in integument and is not visible externally; (3) cartilage lines the inner ventral margin of the clasper glans and often forms the component shield

86. Ventral terminal cartilage (accessory terminal 1 cartilage in rajids): (0) simple; (1) free distally and forms component sentinel or is fused with ventral marginal cartilage and forms component projection; (2) folded ventrally along its long axis to form a convex flange

87. Ventral terminal cartilage (accessory terminal 1 cartilage in rajids): (0) attached over length to axial cartilage; (1) free of axial cartilage

88. Spiracularis: 0 = undivided; (1) splits into lateral and medial bundles, with the medial bundle inserting onto the posterior surface of Meckel’s cartilage and the lateral bundle inserting onto the dorsal edge of the hyomandibula; (2) extends beyond the hyomandibula and Meckel’s cartilage; (3) subdivided proximally and inserts separately onto the palatoquadrate and the hyomandibula.

89. Sexual heterodonty: (0) absent; (1) present.

90. Medial symphyseal processes of the Meckel’s cartilage: (0) absent; (1) present.

91. Lateral processes of the palatoquadrate extending far anteriorly: (0) absent; (1) present.

92. Anterior processes of the Meckel’s cartilage: (0) absent; (1) present; (2) extending anterior past jaw joint.

93. Lateral oral diastema alt: (0) diastema width greater than occlusal width; (1) occlusal width greater than diastema width.

94. Upper jaw profile: (0) oval in cross-section (most batoids); (1) flat top, convex occlusal surface (myliobatids); (2) strongly flattened (mobulids).

95. Upper jaw mineralization: (0) all surfaces mineralized; (1) lingual face partly unmineralized (mobulids).

96. Lower jaw profile: (0) oval in cross-section; (1) strongly linguolabially expanded.

97. Upper and lower jaw trabeculae: (0) absent; (1) weakly developed; (2) strongly developed.

98. Mesiodistally enlarged teeth up to one single tooth row: (0) absent; (1) present.

99. Second transverse keel: (0) absent; (1) present.

100. Calcification pattern of radials: (0) crustal; (1) catenated.

101. Body disc shape: (0) rhombus, quadrangular or oval, with pectoral fins not greatly expanded; (1) wing like, with pectoral fins greatly expanded.

102. Mid-dorsal surface of disc covered by heart-shaped denticles arranged in an antero-posteriorly directed patch having sharply defined outlines: (0) absent; (1) present.

103. File of enlarged ‘caniniform’ teeth in the upper jaw: (0) absent; (1) present.

**Appendix B.** Character matrix.

|  | **1** | **2** | **3** | **4** | **5** | **6** | **7** | **8** | **9** | **10** | **11** | **12** | **13** | **14** | **15** | **16** | **17** | **18** | **19** | **20** | **21** | **22** | **23** | **24** | **25** | **26** | **27** | **28** | **29** | **30** |
| --- | --- | --- | --- | --- | --- | --- | --- | --- | --- | --- | --- | --- | --- | --- | --- | --- | --- | --- | --- | --- | --- | --- | --- | --- | --- | --- | --- | --- | --- | --- |
| *Rhinobatos* | 0 | 0 | 0 | 0 | 0 | 0 | 0 | 0 | 0 | 0 | 0 | 0 | 0 | 0 | 0 | 0 | 0 | 0 | 0 | 0 | 0 | 0 | 0 | 0 | 0 | 0 | 0 | 0 | 0 | 0 |
| *Raja* | 0 | 0 | 0 | 0 | 0 | 0 | 0 | 0 | 0 | 0 | 0 | 0 | 0 | 0 | 0 | 0 | 0 | 0 | 0 | 0 | 0 | 0 | 0 | 0 | 0 | 0 | 0 | 0 | 0 | 0 |
| *Aetobatus* | 0 | 2 | 0 | 1 | 0 | 0 | 1 | 0 | 1 | 1 | 1 | 1 | 1 | 0 | 1 | 1 | 1 | 1 | 3 | 1 | 2 | 0 | 1 | 1 | 1 | ? | 2 | 1 | ? | 0 |
| *Aetomyleus* | 0 | 2 | 0 | 1 | 0 | 0 | 1 | 0 | [01] | 1 | 1 | 1 | 1 | 0 | 1 | ? | 1 | 1 | 3 | 1 | 2 | 0 | 1 | 0 | 1 | 0 | 2 | 1 | 0 | 0 |
| *Asterotrygon* | ? | ? | ? | ? | 0 | 0 | ? | ? | 0 | 0 | 0 | ? | 1 | 1 | 0 | 0 | 0 | 0 | 1 | ? | ? | 1 | ? | ? | 0 | 0 | 0 | 0 | 0 | 0 |
| *Dasyatis* | 0 | 1 | [01] | 1 | 0 | 0 | 0 | 0 | 0 | 0 | 0 | 1 | 1 | 0 | 0 | 0 | 0 | 0 | 2 | 1 | 1 | 1 | 0 | 0 | 1 | 0 | [01] | 0 | 0 | 0 |
| *Gymnura* | 0 | 1 | 0 | 1 | 0 | 0 | 0 | 0 | 0 | 1 | 0 | 1 | 0 | 0 | 0 | 0 | 0 | 0 | 1 | 1 | 1 | 1 | 0 | 0 | 0 | 0 | 1 | 1 | 1 | 0 |
| *Heliobatis* | ? | ? | ? | ? | 0 | 0 | ? | ? | 0 | 0 | 0 | ? | 1 | ? | 0 | 0 | 0 | 0 | ? | ? | ? | ? | ? | ? | 0 | 0 | 0 | 0 | 0 | 0 |
| *Heliotrygon* | 0 | 0 | 2 | 1 | 0 | 0 | 0 | 0 | 0 | 0 | 0 | 1 | 1 | 0 | 0 | 0 | 0 | 0 | 2 | 1 | 1 | 1 | 0 | 0 | 0 | 0 | 0 | 0 | 0 | 1 |
| *Hexatrygon* | 0 | 0 | ? | ? | 0 | 0 | 0 | 0 | 0 | 0 | 0 | 0 | 0 | 0 | 0 | 0 | 0 | 0 | 1 | ? | 0 | 1 | 0 | 0 | 0 | 0 | 0 | 0 | 0 | 0 |
| *Himantura* | 0 | 1 | [01] | 1 | 0 | 0 | 0 | 0 | 0 | 0 | 0 | 1 | 1 | 0 | 0 | 0 | 0 | 0 | 2 | 1 | 1 | 1 | 0 | 0 | 1 | 0 | 0 | 0 | 0 | 0 |
| *Mobula* | 0 | 2 | 0 | 1 | 1 | 1 | 1 | 0 | 1 | 1 | 1 | 1 | 0 | 0 | 1 | 1 | 0 | 1 | 3 | 1 | 2 | 0 | 1 | 1 | 1 | ? | 2 | 0 | ? | 1 |
| *Myliobatis* | 0 | 2 | 0 | 1 | 0 | 0 | 1 | 0 | 0 | 1 | 1 | 1 | 1 | 0 | 1 | [01] | 1 | 1 | 3 | 1 | 2 | 0 | 1 | 0 | 1 | 0 | 1 | 1 | 0 | 0 |
| *Neotrygon* | 0 | 0 | 0 | 1 | 0 | 0 | 0 | 0 | 0 | 0 | 0 | 1 | 1 | 0 | 0 | 0 | 0 | 0 | 2 | 1 | 1 | 1 | 0 | 0 | 1 | 0 | 0 | 0 | 0 | 0 |
| *Paratrygon* | 0 | 0 | 2 | 1 | 0 | 0 | 0 | 0 | 0 | 0 | 0 | 1 | 1 | 0 | 0 | 0 | 0 | 0 | 2 | 1 | 1 | 1 | 0 | 0 | 0 | 0 | 0 | 0 | 0 | 1 |
| *Pastinachus* | 0 | 1 | 0 | 1 | 0 | 0 | 0 | 0 | 0 | 0 | 0 | 1 | 1 | 0 | 0 | 0 | 0 | 0 | 2 | 1 | 1 | 1 | 0 | 0 | 1 | 0 | ? | 0 | 0 | 0 |
| *Plesiobatis* | 0 | 0 | 0 | 1 | 0 | 0 | 0 | 0 | 1 | 0 | 0 | 1 | 1 | 0 | 0 | 0 | 0 | 0 | 1 | 1 | 1 | 0 | 0 | 2 | 0 | 0 | 0 | 0 | 0 | 0 |
| *Plesiotrygon* | 0 | 0 | 1 | 1 | 0 | 0 | 0 | 0 | 0 | 0 | 0 | 1 | 1 | 1 | 0 | 0 | 0 | 0 | 2 | 1 | 1 | 1 | 0 | 1 | 0 | 1 | 0 | 0 | 0 | 1 |
| *Potamotrygon* | 0 | 0 | 1 | 1 | 0 | 0 | 0 | 0 | 0 | 0 | 0 | 1 | 1 | 1 | 0 | 0 | 0 | 0 | 2 | 1 | 1 | 1 | 0 | 1 | 0 | 1 | 0 | 0 | 0 | 1 |
| *Promyliobatis* | ? | ? | ? | ? | 0 | ? | ? | ? | ? | 1 | ? | 1 | ? | ? | ? | ? | ? | ? | 3 | 1 | 2 | 0 | ? | ? | ? | ? | 0 | 1 | 0 | 0 |
| *Protohimantura* | ? | ? | ? | ? | 0 | 0 | ? | ? | ? | 0 | ? | ? | 1 | 0 | 0 | 0 | 0 | 0 | ? | 1 | 1 | 1 | ? | ? | ? | 0 | 0 | 0 | 0 | ? |
| *Pteroplatytrygon* | 0 | 1 | 0 | 1 | 0 | 0 | 0 | 0 | 1 | 0 | 0 | 1 | 1 | 0 | 0 | 0 | 0 | 0 | 2 | 1 | 1 | 1 | 0 | 0 | 1 | 0 | 0 | 0 | 0 | 0 |
| *Rhinoptera* | 0 | 2 | 0 | 1 | 1 | 1 | 1 | 0 | 1 | 1 | 1 | 1 | 1 | 0 | 1 | 1 | 1 | 1 | 3 | 1 | 2 | 0 | 1 | ? | 1 | ? | 2 | 0 | ? | 1 |
| *Styracura* | 0 | 0 | 0 | 1 | 0 | 0 | 0 | 0 | 0 | 0 | 0 | 1 | 1 | 1 | 0 | 0 | 0 | 0 | 2 | 1 | 1 | 1 | 0 | 0 | 1 | 0 | 0 | 0 | 0 | 0 |
| *Taeniura* | 0 | 0 | 0 | 1 | 0 | 0 | 0 | 0 | 0 | 0 | 0 | 1 | 1 | 0 | 0 | 0 | 0 | 0 | 2 | 1 | 1 | 1 | 0 | 0 | 1 | 0 | 0 | 0 | 0 | 0 |
| *Trygonoptera* | 0 | 0 | 0 | 1 | 0 | 0 | 0 | 1 | 0 | 0 | 0 | 1 | 1 | 0 | 0 | 0 | 0 | 0 | 1 | 1 | 1 | 1 | 0 | 0 | 1 | 0 | 0 | 0 | 2 | 0 |
| *Urobatis* | 1 | 0 | 0 | 1 | 0 | 0 | 0 | 0 | 0 | 0 | 0 | 1 | 1 | 0 | 0 | 0 | 0 | 0 | 2 | 1 | 1 | 1 | 0 | [01] | 1 | 0 | 0 | 0 | 0 | 0 |
| *Urolophus* | 0 | 0 | 0 | 1 | 0 | 0 | 0 | 1 | 1 | 0 | 0 | 1 | 1 | 0 | 1 | 0 | 0 | 0 | 1 | 1 | 1 | 1 | 0 | 0 | 0 | 0 | 0 | 0 | 2 | 0 |
| *Urotrygon* | 1 | 0 | 0 | 1 | 0 | 0 | 0 | 0 | 0 | 0 | 0 | 1 | 1 | 0 | 0 | 0 | 0 | 0 | 3 | 1 | 1 | 1 | 0 | 0 | 1 | 0 | 0 | 0 | 0 | 0 |
| *Weissobatis* | ? | ? | ? | ? | 0 | ? | ? | ? | ? | 1 | ? | 1 | ? | ? | ? | ? | ? | ? | ? | ? | ? | ? | ? | ? | ? | ? | 1 | 1 | 0 | 0 |
|  |  |  |  |  |  |  |  |  |  |  |  |  |  |  |  |  |  |  |  |  |  |  |  |  |  |  |  |  |  |  |
|  |  |  |  |  |  |  |  |  |  |  |  |  |  |  |  |  |  |  |  |  |  |  |  |  |  |  |  |  |  |  |
|  |  |  |  |  |  |  |  |  |  |  |  |  |  |  |  |  |  |  |  |  |  |  |  |  |  |  |  |  |  |  |
|  | **31** | **32** | **33** | **34** | **35** | **36** | **37** | **38** | **39** | **40** | **41** | **42** | **43** | **44** | **45** | **46** | **47** | **48** | **49** | **50** | **51** | **52** | **53** | **54** | **55** | **56** | **57** | **58** | **59** | **60** |
| *Rhinobatos* | 0 | 0 | 0 | 0 | 0 | 0 | 0 | 0 | 0 | 0 | 0 | 0 | 0 | 0 | 0 | 0 | 0 | 0 | 0 | ? | ? | ? | 0 | 0 | ? | 0 | 0 | 0 | 0 | 0 |
| *Raja* | 0 | 0 | 0 | 0 | 0 | 0 | 0 | 0 | 0 | 0 | 0 | 0 | 0 | 0 | 0 | 0 | 0 | 0 | 0 | ? | ? | ? | 0 | 0 | ? | 0 | 0 | 0 | 0 | 0 |
| *Aetobatus* | 1 | 0 | 1 | 2 | 1 | 0 | 1 | 1 | 0 | 0 | 0 | 2 | 1 | 1 | 1 | 2 | 1 | ? | ? | 2 | 1 | 2 | 1 | 1 | 2 | 1 | 1 | 0 | 0 | 1 |
| *Aetomyleus* | 0 | 0 | 1 | 2 | 1 | 0 | 1 | 1 | 0 | 0 | 0 | 1 | 1 | ? | ? | 1 | 0 | 1 | 0 | [01] | [01] | [12] | ? | 1 | 1 | ? | 1 | ? | ? | 1 |
| *Asterotrygon* | 0 | 0 | 0 | 1 | ? | ? | ? | ? | ? | ? | ? | 0 | ? | 0 | 0 | 0 | 0 | 0 | 0 | ? | ? | ? | 0 | 0 | ? | 0 | 0 | 0 | 0 | 0 |
| *Dasyatis* | 0 | 1 | 1 | 1 | 0 | 0 | 0 | 0 | 0 | 0 | 0 | 0 | 1 | 0 | 0 | 0 | 0 | 0 | 0 | ? | ? | ? | 0 | 0 | ? | 0 | [12] | 0 | 0 | 0 |
| *Gymnura* | 1 | 1 | 0 | 2 | 0 | 0 | 0 | 0 | 0 | 0 | 0 | 0 | 1 | 0 | 0 | 0 | 0 | 0 | 0 | ? | ? | ? | 0 | 0 | ? | 0 | 0 | 0 | 0 | 0 |
| *Heliobatis* | 0 | 1 | 0 | 1 | ? | ? | ? | ? | ? | ? | ? | 0 | ? | 0 | 0 | 0 | 0 | 0 | 0 | ? | ? | ? | 0 | 0 | ? | 0 | 0 | 0 | 0 | 0 |
| *Heliotrygon* | 0 | 1 | 1 | 2 | 0 | 0 | 0 | 0 | 1 | 1 | 0 | 0 | 1 | ? | ? | 0 | 0 | 0 | 0 | ? | ? | ? | 0 | ? | ? | ? | ? | ? | ? | ? |
| *Hexatrygon* | 0 | 1 | 0 | 0 | 0 | ? | 0 | ? | 0 | 0 | 0 | 0 | 0 | 0 | 0 | 0 | 0 | 0 | 0 | ? | ? | ? | 0 | 0 | ? | 0 | 0 | 0 | 0 | 0 |
| *Himantura* | 0 | 1 | 1 | 2 | 0 | 0 | 0 | 0 | 0 | 0 | 0 | 0 | 1 | 0 | 0 | 0 | 0 | 0 | 0 | ? | ? | ? | 0 | 0 | ? | 0 | 0 | 0 | 0 | 0 |
| *Mobula* | 1 | 0 | 1 | 2 | 1 | 0 | 1 | 1 | 0 | 0 | 0 | 3 | 1 | 0 | 1 | 1 | 0 | [01] | [01] | 0 | ? | 1 | 0 | 2 | ? | 1 | [012] | 0 | 0 | 1 |
| *Myliobatis* | 0 | 0 | 1 | 2 | 1 | 0 | 1 | 1 | 0 | 0 | 0 | 1 | 1 | 1 | 1 | 1 | 0 | 1 | 0 | [01] | [01] | [12] | [01] | 1 | 1 | 0 | 1 | 0 | 0 | 1 |
| *Neotrygon* | 0 | 1 | 1 | 1 | 0 | 1 | 0 | 0 | 0 | 0 | 0 | 0 | 1 | 0 | 0 | 0 | 0 | 0 | 0 | ? | ? | ? | 0 | 0 | ? | 0 | 0 | 0 | 0 | 0 |
| *Paratrygon* | 0 | 1 | 1 | 2 | 0 | 0 | 0 | 0 | 1 | 1 | 0 | 0 | 1 | 0 | 0 | 0 | 0 | 0 | 0 | ? | ? | ? | 0 | 0 | ? | 0 | 0 | 0 | 0 | 0 |
| *Pastinachus* | 0 | 1 | 1 | 1 | 0 | 0 | 0 | 0 | 0 | 0 | 0 | 0 | 1 | ? | ? | 0 | 0 | 0 | 0 | ? | 0 | ? | 0 | 0 | ? | 0 | [12] | ? | ? | 0 |
| *Plesiobatis* | 0 | 1 | 0 | 0 | 0 | 0 | 0 | 0 | 0 | 0 | 0 | 0 | 1 | 0 | 0 | 0 | 0 | 0 | 0 | ? | ? | ? | 0 | 0 | ? | 0 | 0 | 0 | 0 | 0 |
| *Plesiotrygon* | 0 | 1 | 1 | 1 | 0 | 2 | 0 | 0 | 1 | 1 | 0 | 0 | 1 | 0 | 0 | 0 | 0 | 0 | 0 | ? | ? | ? | 0 | 0 | ? | 0 | 0 | 0 | 0 | 0 |
| *Potamotrygon* | 0 | 1 | 1 | 1 | 0 | 2 | 0 | 0 | 1 | 1 | 0 | 0 | 1 | 0 | 0 | 0 | 0 | 0 | 0 | ? | ? | ? | 0 | 0 | ? | 0 | 0 | 0 | 0 | 0 |
| *Promyliobatis* | 0 | ? | 1 | 2 | ? | ? | ? | ? | ? | ? | ? | 1 | ? | 1 | 1 | 1 | 0 | 1 | 0 | 1 | 1 | ? | 1 | ? | ? | ? | 1 | ? | ? | 1 |
| *Protohimantura* | ? | ? | ? | ? | ? | ? | ? | ? | ? | ? | ? | ? | ? | 0 | 0 | 0 | 0 | 0 | 0 | ? | ? | ? | 0 | 0 | ? | 0 | 0 | 0 | 0 | 0 |
| *Pteroplatytrygon* | 0 | 1 | 1 | 1 | 0 | 0 | 0 | 0 | 0 | 0 | 0 | 0 | 1 | 0 | 0 | 0 | 0 | 0 | 0 | ? | ? | ? | 0 | 0 | ? | 0 | 0 | 0 | 0 | 0 |
| *Rhinoptera* | 1 | 0 | 1 | 2 | 1 | 0 | 1 | 1 | 0 | 0 | 0 | 3 | 1 | 1 | 1 | 1 | 0 | 1 | 1 | 0 | 1 | 2 | 0 | 1 | 0 | 0 | 1 | 0 | 0 | 1 |
| *Styracura* | 0 | 1 | 1 | 2 | 0 | 2 | 0 | 0 | 0 | 0 | 0 | 0 | 1 | 0 | 0 | 0 | 0 | 0 | 0 | ? | ? | ? | 0 | 0 | ? | 0 | 0 | 0 | 0 | 0 |
| *Taeniura* | 0 | 1 | 1 | 1 | 0 | 1 | 0 | 0 | 0 | 0 | 0 | 0 | 1 | 0 | 0 | 0 | 0 | 0 | 0 | ? | ? | ? | 0 | 0 | ? | 0 | 0 | 0 | 0 | 0 |
| *Trygonoptera* | 0 | 0 | 0 | 0 | 0 | ? | 0 | 0 | 0 | 0 | 0 | 0 | 1 | 0 | 0 | 0 | 0 | 0 | 0 | ? | ? | ? | 0 | 0 | ? | 0 | 0 | 0 | 0 | 0 |
| *Urobatis* | 0 | 1 | 0 | 0 | 0 | 0 | 0 | 0 | 0 | 0 | 1 | 0 | 1 | 0 | 0 | 0 | 0 | 0 | 0 | ? | ? | ? | 0 | 0 | ? | 0 | 0 | 0 | 0 | 0 |
| *Urolophus* | 0 | [01] | 0 | 0 | 0 | 0 | 0 | 0 | 0 | 0 | 0 | 0 | 1 | 0 | 0 | 0 | 0 | 0 | 0 | ? | ? | ? | 0 | 0 | ? | 0 | 0 | 0 | 0 | 0 |
| *Urotrygon* | 0 | 1 | 0 | 0 | 0 | 0 | 0 | 0 | 0 | 0 | 1 | 0 | 1 | 0 | 0 | 0 | 0 | 0 | 0 | ? | ? | ? | 0 | 0 | ? | 0 | 0 | 0 | 0 | 0 |
| *Weissobatis* | 0 | 0 | 1 | 2 | ? | ? | ? | ? | ? | ? | ? | 1 | ? | 1 | 1 | 1 | 0 | 1 | 0 | 1 | 1 | 2 | 1 | 1 | 1 | 0 | 1 | ? | 0 | 1 |
|  |  |  |  |  |  |  |  |  |  |  |  |  |  |  |  |  |  |  |  |  |  |  |  |  |  |  |  |  |  |  |
|  |  |  |  |  |  |  |  |  |  |  |  |  |  |  |  |  |  |  |  |  |  |  |  |  |  |  |  |  |  |  |
|  |  |  |  |  |  |  |  |  |  |  |  |  |  |  |  |  |  |  |  |  |  |  |  |  |  |  |  |  |  |  |
|  | **61** | **62** | **63** | **64** | **65** | **66** | **67** | **68** | **69** | **70** | **71** | **72** | **73** | **74** | **75** | **76** | **77** | **78** | **79** | **80** | **81** | **82** | **83** | **84** | **85** | **86** | **87** | **88** | **89** | **90** |
| *Rhinobatos* | 0 | 0 | 0 | 0 | ? | 0 | 0 | 0 | 0 | 2 | 0 | 0 | 0 | 0 | 0 | 0 | 0 | 0 | 0 | 0 | 1 | 0 | 0 | 0 | 0 | 0 | 0 | 0 | 0 | 0 |
| *Raja* | 0 | 0 | 0 | 0 | ? | 0 | 0 | 1 | 0 | 0 | 0 | 0 | 0 | 0 | 0 | 0 | 1 | 0 | 0 | 1 | 0 | 1 | 0 | 0 | 3 | 1 | 1 | 0 | 0 | 0 |
| *Aetobatus* | 1 | 2 | 0 | 2 | 0 | 1 | 1 | 2 | 1 | 3 | 2 | 1 | 1 | 1 | 1 | 3 | 2 | 1 | 1 | 1 | 3 | 0 | 1 | 0 | 2 | 2 | 0 | ? | 1 | 0 |
| *Aetomyleus* | 1 | 1 | 0 | ? | ? | ? | [01] | [12] | 0 | ? | 2 | ? | 1 | ? | ? | ? | 2 | 1 | 1 | 1 | ? | ? | ? | ? | ? | ? | ? | ? | 0 | 0 |
| *Asterotrygon* | 0 | 0 | 0 | 0 | ? | ? | 1 | 1 | 0 | ? | ? | ? | 1 | 1 | 1 | 0 | 2 | 1 | 1 | 1 | ? | ? | ? | ? | ? | ? | ? | ? | 1 | ? |
| *Dasyatis* | 0 | 0 | 1 | 0 | ? | 1 | 1 | [12] | 0 | 3 | 1 | [12] | 1 | 1 | 1 | 1 | 2 | 1 | 1 | 1 | 3 | 1 | 0 | 1 | 1 | 2 | 1 | 1 | 1 | 1 |
| *Gymnura* | 0 | 0 | 0 | 0 | ? | 1 | 1 | [12] | 1 | 1 | 0 | 1 | 1 | 1 | 1 | 0 | 2 | 1 | 1 | 1 | 3 | 0 | 1 | 0 | 2 | 2 | 0 | ? | 0 | 0 |
| *Heliobatis* | 0 | 0 | 0 | 0 | ? | ? | 1 | 1 | 0 | ? | ? | ? | 1 | 1 | 1 | 0 | 2 | 1 | 1 | 1 | ? | ? | ? | ? | ? | ? | ? | ? | ? | ? |
| *Heliotrygon* | ? | ? | ? | ? | ? | ? | ? | ? | ? | ? | ? | ? | ? | ? | ? | ? | ? | ? | ? | ? | ? | ? | ? | ? | ? | ? | ? | ? | 0 | 0 |
| *Hexatrygon* | 0 | 0 | 0 | 0 | ? | 1 | 1 | 2 | 1 | 1 | 0 | ? | 1 | 1 | 1 | 0 | 2 | 1 | 1 | 1 | 2 | ? | ? | ? | ? | ? | ? | ? | 0 | 0 |
| *Himantura* | 0 | 0 | 0 | 0 | ? | 1 | 1 | 1 | 0 | 1 | 0 | 2 | 1 | 1 | 1 | 1 | 2 | 1 | 1 | 1 | 3 | 0 | 1 | 0 | 1 | 2 | 1 | 2 | 1 | 1 |
| *Mobula* | 1 | ? | 0 | 0 | [01] | 1 | 1 | [12] | 1 | ? | [01] | 1 | 1 | 1 | 1 | 3 | 2 | 1 | 1 | 1 | ? | 1 | ? | ? | ? | ? | ? | ? | [01] | 0 |
| *Myliobatis* | 1 | 1 | [01] | 0 | 0 | 1 | 1 | 2 | 1 | 3 | 2 | 1 | 1 | 1 | 1 | 3 | 2 | 1 | 1 | 1 | 3 | 0 | 1 | 0 | 2 | 2 | 0 | 0 | 0 | 0 |
| *Neotrygon* | 0 | 0 | 0 | 0 | ? | 0 | 1 | [12] | [01] | 1 | 0 | 1 | 1 | 1 | 0 | 1 | 2 | 1 | 1 | 1 | 2 | 0 | 0 | 1 | 1 | 2 | 1 | 2 | 1 | 0 |
| *Paratrygon* | 0 | 0 | 0 | 0 | ? | 1 | 1 | 1 | 0 | ? | 0 | ? | 1 | 1 | 1 | 1 | 2 | 1 | 1 | 1 | ? | 0 | ? | ? | ? | ? | ? | 2 | 0 | 0 |
| *Pastinachus* | 0 | 0 | 0 | ? | ? | ? | 1 | 1 | 1 | ? | 2 | ? | 1 | ? | ? | ? | 2 | 1 | 1 | 1 | ? | ? | ? | ? | ? | ? | ? | ? | 0 | 0 |
| *Plesiobatis* | 0 | 0 | 0 | 0 | ? | 1 | 1 | 1 | 1 | ? | 0 | 1 | 1 | 1 | 1 | 0 | 2 | 1 | 1 | 1 | 1 | 0 | 1 | 0 | ? | 2 | 0 | 1 | 0 | 0 |
| *Plesiotrygon* | 0 | 0 | 0 | 0 | ? | 1 | 1 | 1 | 0 | ? | 1 | ? | 1 | 1 | 1 | 1 | 2 | 1 | 1 | 1 | ? | 0 | ? | ? | ? | ? | ? | 2 | 0 | 0 |
| *Potamotrygon* | 0 | 0 | 0 | 0 | ? | 1 | 1 | 1 | 0 | 1 | 1 | 3 | 1 | 1 | 1 | 1 | 2 | 1 | 1 | 1 | 2 | 0 | 1 | 0 | 1 | 2 | 0 | 2 | 0 | 0 |
| *Promyliobatis* | 1 | 1 | 1 | ? | 0 | ? | 1 | 2 | 1 | ? | ? | ? | 1 | ? | ? | 3 | ? | ? | 1 | 1 | 3 | ? | ? | ? | ? | ? | ? | ? | ? | ? |
| *Protohimantura* | 0 | 0 | 0 | 0 | ? | ? | ? | 1 | ? | ? | ? | ? | 1 | 1 | ? | ? | 2 | ? | 1 | 1 | 3 | ? | ? | ? | ? | ? | ? | ? | ? | ? |
| *Pteroplatytrygon* | 0 | 0 | 0 | 0 | ? | 1 | 1 | 1 | 0 | ? | 1 | 1 | 1 | 1 | 1 | 1 | 2 | 1 | 1 | 1 | 3 | 1 | 0 | 1 | 1 | 2 | 1 | 1 | 1 | 0 |
| *Rhinoptera* | 1 | 2 | 1 | 1 | 0 | 1 | 1 | 2 | 1 | 3 | 2 | 1 | 1 | 1 | 1 | 3 | 2 | 1 | 1 | 1 | ? | 0 | 1 | 0 | 2 | 2 | 0 | 3 | ? | ? |
| *Styracura* | 0 | 0 | 0 | 0 | ? | 1 | 1 | 1 | 0 | ? | ? | 1 | 1 | 1 | 1 | 1 | 2 | 1 | 1 | 1 | 2 | ? | ? | ? | ? | ? | ? | ? | 0 | 0 |
| *Taeniura* | 0 | 0 | 0 | 0 | ? | 1 | 1 | [12] | 0 | 3 | 1 | 1 | 1 | 1 | 1 | 1 | 2 | 1 | 1 | 1 | 2 | 0 | 0 | 1 | 1 | 2 | 1 | 2 | 1 | 0 |
| *Trygonoptera* | 0 | 0 | 0 | 0 | ? | 1 | 1 | 2 | 1 | ? | 0 | ? | 1 | 1 | 1 | ? | 2 | 1 | 1 | 1 | ? | 0 | ? | ? | ? | ? | ? | ? | 0 | 0 |
| *Urobatis* | 0 | 0 | 0 | 0 | ? | 1 | 1 | [12] | 0 | ? | 0 | 1 | 1 | 1 | 1 | 1 | 2 | 1 | 1 | 1 | 1 | 0 | 1 | 0 | 2 | 2 | 0 | 1 | 0 | 0 |
| *Urolophus* | 0 | 0 | 0 | 0 | ? | 1 | 1 | 2 | 1 | 1 | 0 | 1 | 1 | 1 | 1 | 0 | 2 | 1 | 1 | 1 | 1 | 0 | 1 | 0 | 2 | 2 | 0 | 1 | 1 | 0 |
| *Urotrygon* | 0 | 0 | 0 | 0 | ? | 1 | 1 | [12] | 0 | 1 | 0 | 1 | 1 | 1 | 1 | 2 | 2 | 1 | 1 | 1 | 1 | 0 | 1 | 0 | 2 | 2 | 0 | 1 | 0 | 0 |
| *Weissobatis* | 1 | 1 | 0 | 0 | 0 | ? | 1 | 2 | 1 | ? | ? | ? | 1 | ? | ? | ? | 2 | ? | 1 | 1 | ? | ? | ? | ? | ? | ? | ? | ? | ? | ? |
|  |  |  |  |  |  |  |  |  |  |  |  |  |  |  |  |  |  |  |  |  |  |  |  |  |  |  |  |  |  |  |
|  |  |  |  |  |  |  |  |  |  |  |  |  |  |  |  |  |  |  |  |  |  |  |  |  |  |  |  |  |  |  |
|  |  |  |  |  |  |  |  |  |  |  |  |  |  |  |  |  |  |  |  |  |  |  |  |  |  |  |  |  |  |  |
|  | **91** | **92** | **93** | **94** | **95** | **96** | **97** | **98** | **99** | **100** | **101** | **102** | **103** |  |  |  |  |  |  |  |  |  |  |  |  |  |  |  |  |  |
| *Rhinobatos* | 0 | 0 | 0 | 0 | 0 | 0 | 0 | 0 | 0 | 0 | 0 | 0 | 0 |  |  |  |  |  |  |  |  |  |  |  |  |  |  |  |  |  |
| *Raja* | 0 | 0 | 0 | 0 | 0 | 0 | 0 | 0 | 0 | 1 | 0 | 0 | 0 |  |  |  |  |  |  |  |  |  |  |  |  |  |  |  |  |  |
| *Aetobatus* | 1 | 0 | 0 | 0 | 0 | 1 | 1 | 1 | 0 | 0 | 1 | 0 | 0 |  |  |  |  |  |  |  |  |  |  |  |  |  |  |  |  |  |
| *Aetomyleus* | 0 | 2 | 0 | 1 | 0 | 1 | 1 | 1 | 0 | 0 | 1 | 0 | 0 |  |  |  |  |  |  |  |  |  |  |  |  |  |  |  |  |  |
| *Asterotrygon* | ? | ? | ? | ? | ? | ? | ? | 0 | 0 | 1 | 0 | 0 | 0 |  |  |  |  |  |  |  |  |  |  |  |  |  |  |  |  |  |
| *Dasyatis* | 0 | 2 | 0 | 0 | 0 | 0 | 0 | 0 | 0 | 1 | 0 | 0 | 0 |  |  |  |  |  |  |  |  |  |  |  |  |  |  |  |  |  |
| *Gymnura* | 0 | 0 | 1 | 0 | 0 | 0 | 0 | 0 | 0 | 0 | 1 | 0 | 0 |  |  |  |  |  |  |  |  |  |  |  |  |  |  |  |  |  |
| *Heliobatis* | ? | ? | ? | ? | ? | ? | ? | 0 | 0 | 1 | 0 | 0 | 0 |  |  |  |  |  |  |  |  |  |  |  |  |  |  |  |  |  |
| *Heliotrygon* | 0 | 0 | 0 | 0 | 0 | 0 | 0 | ? | ? | ? | 0 | 0 | 0 |  |  |  |  |  |  |  |  |  |  |  |  |  |  |  |  |  |
| *Hexatrygon* | 0 | 0 | 0 | 0 | 0 | 0 | 0 | 0 | 0 | 1 | 0 | 0 | 0 |  |  |  |  |  |  |  |  |  |  |  |  |  |  |  |  |  |
| *Himantura* | 0 | 2 | 0 | 0 | 0 | 0 | 0 | 0 | 1 | 1 | 0 | 1 | 0 |  |  |  |  |  |  |  |  |  |  |  |  |  |  |  |  |  |
| *Mobula* | 0 | 0 | 1 | 2 | 1 | 1 | 2 | 1 | 0 | 0 | 1 | 0 | 0 |  |  |  |  |  |  |  |  |  |  |  |  |  |  |  |  |  |
| *Myliobatis* | 1 | 2 | 0 | 1 | 0 | 1 | 1 | 1 | 0 | 0 | 1 | 0 | 0 |  |  |  |  |  |  |  |  |  |  |  |  |  |  |  |  |  |
| *Neotrygon* | 0 | 1 | 0 | 0 | 0 | 0 | 0 | 0 | 0 | ? | 0 | 0 | 1 |  |  |  |  |  |  |  |  |  |  |  |  |  |  |  |  |  |
| *Paratrygon* | 0 | 0 | 1 | 0 | 0 | 0 | 0 | 0 | 0 | 1 | 0 | 0 | 0 |  |  |  |  |  |  |  |  |  |  |  |  |  |  |  |  |  |
| *Pastinachus* | 0 | 0 | 0 | 0 | 0 | 1 | 0 | 1 | 0 | 1 | 0 | 0 | 0 |  |  |  |  |  |  |  |  |  |  |  |  |  |  |  |  |  |
| *Plesiobatis* | 0 | 0 | 0 | 0 | 0 | 0 | 0 | 0 | 0 | 0 | 0 | 0 | 0 |  |  |  |  |  |  |  |  |  |  |  |  |  |  |  |  |  |
| *Plesiotrygon* | 0 | 0 | 0 | 0 | 0 | 0 | 0 | 0 | 0 | 1 | 0 | 0 | 0 |  |  |  |  |  |  |  |  |  |  |  |  |  |  |  |  |  |
| *Potamotrygon* | 0 | 0 | 0 | 0 | 0 | 0 | 0 | 0 | 0 | 1 | 0 | 0 | 0 |  |  |  |  |  |  |  |  |  |  |  |  |  |  |  |  |  |
| *Promyliobatis* | ? | ? | ? | ? | ? | ? | ? | 1 | 0 | 0 | 1 | 0 | 0 |  |  |  |  |  |  |  |  |  |  |  |  |  |  |  |  |  |
| *Protohimantura* | ? | ? | 0 | ? | 0 | ? | ? | 0 | 1 | 1 | 0 | 1 | 0 |  |  |  |  |  |  |  |  |  |  |  |  |  |  |  |  |  |
| *Pteroplatytrygon* | 0 | 0 | 0 | 0 | 0 | 0 | 0 | 0 | 0 | 1 | 0 | 0 | 0 |  |  |  |  |  |  |  |  |  |  |  |  |  |  |  |  |  |
| *Rhinoptera* | 0 | 0 | ? | 1 | 0 | 1 | 1 | 1 | 0 | 0 | 1 | 0 | 0 |  |  |  |  |  |  |  |  |  |  |  |  |  |  |  |  |  |
| *Styracura* | 0 | ? | 0 | 0 | 0 | 0 | 0 | 0 | 0 | 1 | 0 | 0 | 0 |  |  |  |  |  |  |  |  |  |  |  |  |  |  |  |  |  |
| *Taeniura* | 0 | 1 | 0 | 0 | 0 | 0 | 0 | 0 | 0 | 1 | 0 | 0 | 1 |  |  |  |  |  |  |  |  |  |  |  |  |  |  |  |  |  |
| *Trygonoptera* | 0 | 1 | 0 | 0 | 0 | 0 | 0 | 0 | 1 | 1 | 0 | 0 | 0 |  |  |  |  |  |  |  |  |  |  |  |  |  |  |  |  |  |
| *Urobatis* | 0 | 0 | 0 | 0 | 0 | 0 | 0 | 0 | 1 | 1 | 0 | 0 | 0 |  |  |  |  |  |  |  |  |  |  |  |  |  |  |  |  |  |
| *Urolophus* | 0 | 0 | 0 | 0 | 0 | 0 | 0 | 0 | 1 | 1 | 0 | 0 | 0 |  |  |  |  |  |  |  |  |  |  |  |  |  |  |  |  |  |
| *Urotrygon* | 0 | 0 | 0 | 0 | 0 | 0 | 0 | 0 | 0 | 1 | 0 | 0 | 0 |  |  |  |  |  |  |  |  |  |  |  |  |  |  |  |  |  |
| *Weissobatis* | ? | ? | ? | ? | ? | ? | ? | 1 | 0 | 0 | 1 | 0 | 0 |  |  |  |  |  |  |  |  |  |  |  |  |  |  |  |  |  |

**Supplementary References**

Aschliman NC. Interrelationships of the durophagous stingrays (Batoidea: Myliobatidae). Environ Biol Fishes. 2014; 97: 967-979.

Claeson KM, O’Leary MA, Roberts EM, Sissoko F, Bouaré M, Tapanila L, Goodwin D, Gottfried MD. First Mesozoic record of the stingray *Myliobatis wurnoensis* from Mali and a phylogenetic analysis of Myliobatidae incorporating dental characters. Acta Palaeontol Pol. 2010; 55:655-674.

Hovestadt DC, Hovestadt-Euler M. *Weissobatis micklichi* n. gen., n. sp., an eagle ray (Myliobatiformes, Myliobatidae) from the Oligocene of Frauenweiler (Baden-Württemberg, Germany). Paläont Z. 1999; 73:337-349.

Hovestadt DC, Hovestadt-Euler M. Generic assessment and reallocation of Cenozoic myliobatins based on new information of tooth, tooth plate and caudal spine morphology of extant taxa. Palaeontos. 2013; 24:1-66.

Marramà G, Bannikov AF, Tyler JC, Zorzin R, Carnevale G. Controlled excavations in the Pesciara and Monte Postale sites provide new insights about the paleoecology and taphonomy of the fish assemblages of the Eocene Bolca Konservat-Lagerstätte, Italy. Palaeogeogr Palaeoclimat Palaeoecol. 2016; 454:228-245.

Marramà G, Carnevale G, Naylor JP, Kriwet J. Reappraisal of the Eocene whiptail stingrays (Myliobatiformes, Dasyatidae) of the Bolca Lagerstätte, Italy. Zool Scr. 2018a. doi:10.1111/zsc.12330.

Marramà G, Klug S, De Vos J, Kriwet J. Anatomy, relationships and palaeobiogeographic implications of the first Neogene holomorphic stingray (Myliobatiformes: Dasyatidae) from the early Miocene of Sulawesi, Indonesia, SE Asia. Zool J Linn Soc. 2018b. doi:10.1093/zoolinnean/zly020.

Papazzoni CA, Trevisani E. Facies analysis, palaeoenvironmental reconstruction, and biostratigraphy of the “Pesciara di Bolca” (Verona, northern Italy): An early Eocene Fossil- Lagerstätte. Palaeogeogr Palaeoclimatol Palaeoecol. 2006; 242:21-35.

Papazzoni CA, Carnevale G, Fornaciari E, Giusberti L, Trevisani E. The Pesciara-Monte Postale Fossil-Lagerstätte: 1. Biostratigraphy, sedimentology and depositional model. In: Papazzoni CA, Giusberti L, Carnevale G, Roghi G, Bassi D, Zorzin R, editors. The Bolca Fossil-Lagerstätte: A window into the Eocene World. Modena: Società Paleontologica Italiana; 2014. p. 29-36.

Trevisani E. Upper Cretaceous‑Lower Eocene succession of the Monte Postale and its relationship with the “Pesciara di Bolca” (Lessini Mountains, northern Italy): deposition of a fossil‑fish Lagerstätte. Facies. 2015; 61:1-17.

White WT. A revised generic arrangement for the eagle ray family Myliobatidae, with definitions for the valid genera. Zootaxa. 2014; 3860:149-166.
